# PLK1 Regulates MicroRNA Biogenesis through Drosha Phosphorylation

**DOI:** 10.3390/ijms241814290

**Published:** 2023-09-19

**Authors:** Claire Emily Fletcher, Molly Ann Taylor, Charlotte Lynne Bevan

**Affiliations:** 1 Department of Surgery & Cancer, Imperial College London, Hammersmith Hospital, Du Cane Road, London W12 0NN, UK; 2 Oncology R&D, AstraZeneca, Cambridge CB2 0AA, UK

**Keywords:** microRNA, PLK1, Drosha, phosphorylation, microRNA biogenesis, non-coding RNA, kinase

## Abstract

Polo-Like Kinase 1 (PLK1), a key mediator of cell-cycle progression, is associated with poor prognosis and is a therapeutic target in a number of malignancies. Putative phosphorylation sites for PLK1 have been identified on Drosha, the main catalytic component of the microprocessor responsible for miR biogenesis. Several kinases, including GSK3β, p70 S6 kinase, ABL, PAK5, p38 MAPK, CSNK1A1 and ANKRD52-PPP6C, have been shown to phosphorylate components of the miR biogenesis machinery, altering their activity and/or localisation, and therefore the biogenesis of distinct miR subsets. We hypothesised that PLK1 regulates miR biogenesis through Drosha phosphorylation. In vitro kinase assays confirmed PLK1 phosphorylation of Drosha at S^300^ and/or S^302^. PLK1 inhibition reduced serine-phosphorylated levels of Drosha and its RNA-dependent association with DGCR8. In contrast, a “phospho-mimic” Drosha mutant showed increased association with DGCR8. PLK1 phosphorylation of Drosha alters Drosha Microprocessor complex subcellular localisation, since PLK1 inhibition increased cytosolic protein levels of both DGCR8 and Drosha, whilst nuclear levels were decreased. Importantly, the above effects are independent of PLK1’s cell cycle-regulatory role, since altered Drosha:DGCR8 localisation upon PLK1 inhibition occurred prior to significant accumulation of cells in M-phase, and PLK1-regulated miRs were not increased in M-phase-arrested cells. Small RNA sequencing and qPCR validation were used to assess downstream consequences of PLK1 activity on miR biogenesis, identifying a set of ten miRs (miR-1248, miR-1306-5p, miR-2277-5p, miR-29c-5p, miR-93-3p, miR-152-3p, miR-509-3-5p, miR-511-5p, miR-891a-5p and miR-892a) whose expression levels were statistically significantly downregulated by two pharmacological PLK1 kinase domain inhibitors, RO-5203280 and GSK461364. Opposingly, increased levels of these miRs were observed upon transfection of wild-type or constitutively active PLK1. Importantly, pre-miR levels were reduced upon PLK1 inhibition, and pri-miR levels decreased upon PLK1 activation, and hence, PLK1 Drosha phosphorylation regulates MiR biogenesis at the level of pri-miR-to-pre-miR processing. In combination with prior studies, this work identifies Drosha S^300^ and S^302^ as major integration points for signalling by several kinases, whose relative activities will determine the relative biogenesis efficiency of different miR subsets. Identified kinase-regulated miRs have potential for use as kinase inhibitor response-predictive biomarkers, in cancer and other diseases.

## 1. Introduction

MiRs are ~22 nucleotide non-coding RNAs that regulate gene expression through association with target gene mRNAs, often at the 3′UTR. A single transcript can be targeted by several miRs, and individual miRs can target hundreds of genes, resulting in complex networks of tissue- and disease-specific interactions. Small RNA-based therapeutics show considerable promise in a wide range of human pathologies due to their target specificity, stability, small size and potential for tissue-specific targeting. Indeed, six anti-sense oligonucleotides (ASOs) are FDA-approved for a range of conditions, with three seeking regulatory approval [1]. MiRs also have considerable potential as non-invasive diagnostic, prognostic and predictive biomarkers [2]. Mechanisms governing the complex, multi-step biogenesis of miRs are poorly understood. In the first critical step, primary miR transcripts (pri-miRs) are cleaved into ~70 nt hairpin-structured precursor-miRs (pre-miRs) by the Drosha-containing Microprocessor (MP) complex [3,4,5]. Multiple cofactors determine MP fidelity, efficiency and specificity, altering biogenesis of distinct miR subsets [6,7,8,9]. Coordinated regulation likely prevents spatial/temporal mis-expression of miRs, maintaining their complex post-transcriptional control of gene activity. MiR processing can be altered in both development and disease. During development, many pri-miRs are inefficiently converted into their mature forms [10], while reduced processing contributes to widespread miR downregulation in cancer [11,12]. It is vital to elucidate regulatory processes governing miR biogenesis, and perturbation in disease states, in order to fully exploit miRs as disease biomarkers and therapeutic targets.

We previously identified GSK3β as a key modulator of global miR biogenesis, acting as a component of the MP and RNase cofactor: GSK3β binds to p72 and DGCR8 in the MP in an RNA-dependent manner, leading it to phosphorylate Drosha at S^300^ and S^302^. This phosphorylation increases Drosha association with cofactors and pri-miRs and enhances its RNase and pri-miR cleavage activities, thus increasing mature miR levels [13]. Thus, GSK3β constitutes a vital link between essential mitogenic signalling-pathways and miR biogenesis. In addition, multiple mechanistically undescribed Drosha phosphorylation sites have been identified, and other miR biogenesis effectors are also phosphorylated. For example, p70 S6 kinase phosphorylates Dicer cofactor TTP to inhibit maturation of a miR subset [14], casein kinase II enhances miR biogenesis and function through phosphorylation of RISC component, CGH-1 [15], whilst ABL phosphorylates Drosha partner DGCR8 at Y^267^ to enhance selective pri-miR processing [16]. Recent studies showed cyclical multi-site phosphorylation/dephosphorylation of RISC protein, AGO2, by CSNK1A1 and ANKRD52-PPP6C, respectively [17]. Given the many critical roles of kinases across human diseases, and clinical use of kinase inhibitors, it is noteworthy that their impacts on miR biosynthesis have not been further explored.

Polo-like kinase 1 (PLK1) is a key facilitator of cell-cycle progression, with essential roles in chromosome segregation and cell division [18,19]. PLK1 is required for release from DNA-damage-induced G_2_-phase arrest before mitotic entry [20]. During mitotic entry, PLK1 amplifies cyclin-dependent kinase 1 (CDK1) activation, enabling efficient onset of mitosis [21]. It also mediates centrosome maturation and the accumulation of γ-tubulin complexes on centrosomes [22,23]. In prometaphase, PLK1 is required for the generation of stable kinetochore microtubule attachments [24,25]. PLK1 also promotes dissociation of cohesin from chromosome arms, in prophase and prometaphase, by phosphorylating cohesin’s STAG2 subunit [26], as well as multiple aspects of cytokinesis by phosphorylating activators and effectors of RhoA [27,28]. Multiple PLK1 phosphorylation sites have been identified on Drosha, DGCR8 and DDX5 through phospho-proteomics [29,30]. In particular, Drosha amino acids S^300^, S^373^ and S^807^ were identified as PLK1-phosphorylated in HeLa cells [29,30]. However, functional implications for miR biogenesis regulation were not assessed. We hypothesised that PLK1 may phosphorylate Drosha to modulate biogenesis of a subset of miRs. Herein we demonstrate that PLK1 increases mature levels of a number of miRs by interacting with Drosha in an RNA-dependent manner and phosphorylating it at S^300^ and/or S^302^. This increases Drosha:DGCR8 interaction, enhances Drosha and DGCR8 subcellular localisation, and increases pri-miR-to-pre-miR processing. Importantly, this appears independent of PLK1’s cell cycle regulatory role.

## 2. Results

**Figure 1:**
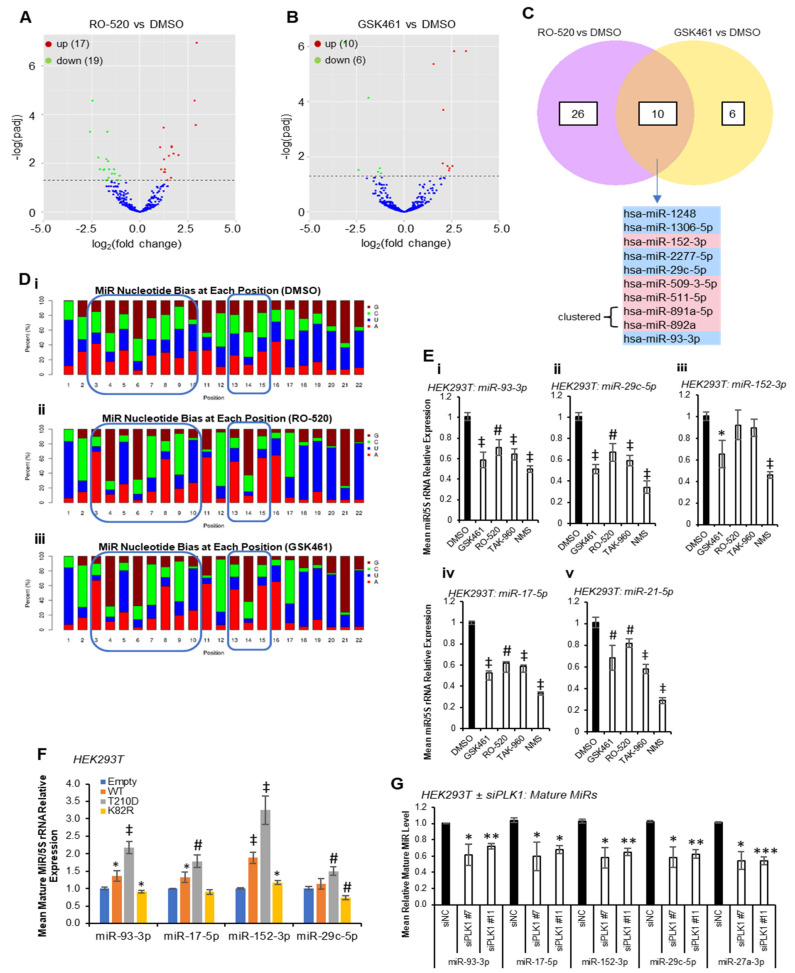
**PLK1 modulates mature microRNA levels**. (**A**,**B**) Volcano plots showing miRs identified via small RNA-seq as significantly dysregulated in HEK293T cells treated with RO-520 (100 nM) or GSK461 (100 nM) for 72 h (n = 3). Green dots—significantly downregulated microRNAs vs. DMSO, red dots—significantly upregulated microRNAs vs. DMSO. (**C**) Pie chart showing miRs dysregulated by both RO-520 and GSK461. (**D**) Nucleotide bias at each microRNA base position for identified RO-520- and GSK461-regulated miRs. (**i**) DMSO, (**ii**) RO-520, (**iii**) GSK461. Blue boxes highlight canonical seed region and additionally 3’ complementarity region. (**E**) qRT-PCR analysis of miR-93-3p (**i**), miR-29c-5p (**ii**), miR-152-3p (**iii**), miR-17-5p (**iv**) and miR-21-5p (**v**) in HEK293T cells treated with GSK461 (100 nM), RO-520 (100 nM), TAK960 (100 nM) and NMS (500 nM) for 72 h. Expression was normalised to 5S rRNA. (**F**) qRT-PCR analysis of miR-93-3p*,* miR-17-5p, miR-152-3p and miR-29c-5p in HEK293T cells transfected with WT, constitutively active (T^210^D) and kinase-dead dominant-negative (K^82^R) PLK1 for 72 h. Expression was normalised to 5S rRNA. (**G**) qRT-PCR analysis of miR-93-3p*,* miR-17-5p, miR-152-3p, miR-29c-5p and miR-27a-3p in HEK293T cells transfected with two different PLK1 siRNAs or negative control siRNA (siNC) (all 15 nM) for 72 h. Expression was normalised to 5S rRNA. (**F**,**G**) *Columns:* mean relative miR levels ± SEM for three independent experiments performed in triplicate. (**E**,**F**) * *p <* 0.05*,* # *p* < 0.001, and ‡ *p* < 0.0001, (**G**) * *p <* 0.05, ** *p* < 0.001, and *** *p* < 0.0001. See also Figures S1–S5, Tables S1 and S2.

Multiple PLK1 phosphorylation sites have been identified within protein components of the miR biogenesis cascade. To identify whether PLK1 modulates miR synthesis, we performed small RNA sequencing (smRNA-seq) on HEK293T cells treated ± PLK1 inhibitors RO-5203280 (RO-520) and GSK461364 (GSK461) for 72 h. These inhibitors were chosen for their selectivity for PLK1 over related kinases. Inhibitor activity was confirmed via Western blotting for phospho-TCTP(Ser^46^)—a well-characterised downstream PLK1 target in HEK293T and MCF7 cells (Figure S1). Here, 17 and 10 mature miRs were found to be significantly upregulated by RO-520 and GSK461, respectively, with 19 and 6 significantly downregulated (Figure 1A,B, Tables S1 and S2). Five miRs were significantly downregulated (FDR ≤ 0.05) by both inhibitors (miR-1248, miR-1306-5p, miR-2277-5p, miR-29c-5p and miR-93-3p), with a further five being significantly increased via both treatments (miR-152-3p, miR-509-3-5p, miR-511-5p, miR-891a-5p and miR-892a) (Figure 1C). Notably, many sequencing reads mapped to non-miR small RNAs, suggesting that other small RNA species may be altered via PLK1 inhibitor treatment (Figure S2).

Nucleotide bias analysis revealed that PLK1i-regulated miRs show altered nucleotide composition compared to DMSO controls, especially in seed regions (Figure 1D), suggesting that PLK1-regulated miRs may converge on targets containing miR recognition elements (MREs) with high sequence similarity. Compared to DMSO-treated cells, the miR population in RO-520- and GSK461-treated cells showed increased prevalence of A at position 3, G at position 4, U at position 5, G at position 6, C at position 7, A at position 8 and U at position 10. There was also increased prevalence of A at positions 15 and 16, and higher frequency of U across 18–20 (Figure 1D). qRT-PCR validation confirmed significant downregulation of RO-520- and GSK461-downregulated miR-93-3p and -29c-5p by an expanded panel of PLK1 inhibitors (Figure 1E(i,ii)). MiR-152-3p (shown to be upregulated by RO-520 and GSK461 via small RNA-seq) was significantly downregulated by GSK461 and NMS1286937 (NMS) via qRT-PCR (Figure 1E(iii)). MiR-17-5p (downregulated by RO-520 in small RNA-seq data) was significantly reduced by all inhibitors via qRT-PCR (Figure 1E(iv)). Further, miR-21-5p, which is highly expressed and cancer-implicated was also significantly downregulated via all treatments (Figure 1E(v)). In keeping with the above, transfection of HEK293T cells with plasmids overexpressing WT or constitutively active PLK1(T^210^D) significantly increased levels of validated PLK1-regulated miRs, whilst dominant-negative PLK1(K^82^R) significantly reduced levels of mature miRs 93-3p and -29c-5p, with a modest increase in miR-152-3p observed (Figure 1F). In further corroboration, PLK1 knock-down using siRNAs significantly reduced levels of validated PLK1-regulated miRs (Figure 1G and Figure S3). Pathway analysis of shared RO-520- and GSK461-regulated miRs revealed enrichment for AKT pathway activity, NOTCH signalling, TP53 network, MET activity, senescence, cell cycle progression and TGFβ signalling (Figure S4). Supportive of the cancer relevance of potential PLK1-enhanced miR biogenesis, the most robustly PLK1 inhibitor-downregulated miRs, miR-93-3p, miR-21-5p and miR-17-5p, or their cluster-partner miRs were amongst the top ten PLK1-correlated miRs in 12, 8 and 15 of the 33 cancers of the TCGA data set, respectively (Figure S5).

**Figure 2:**
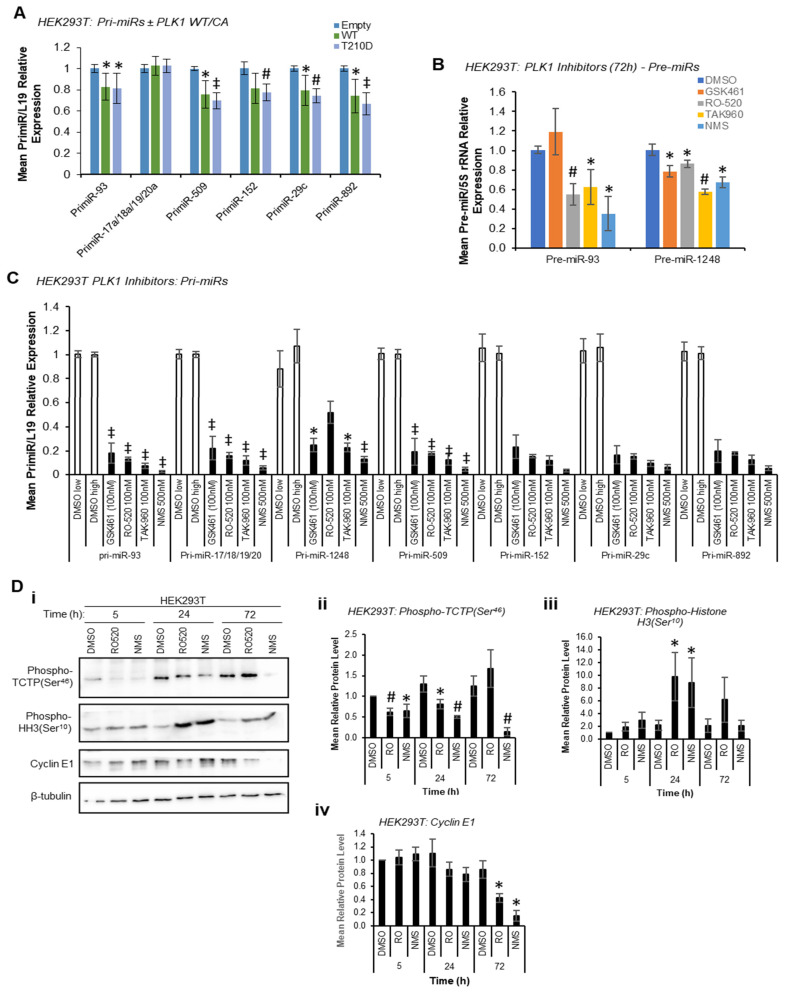
**PLK1 modulates pri-miR and pre-miR levels, and PLK1 inhibitor induces cell cycle M-Phase arrest at 24 h**. (**A**) qRT-PCR analysis of pri-miRs of RNA-seq-identified PLK1-regulated miRs in HEK293T cells following transfection with WT or phospho-mimic (T^210^D) PLK1 for 72 h. Expression was normalised to L19. n = 3. (**B**) qRT-PCR analysis of pre-miR levels in HEK293T cells treated with GSK461 (100 nM), RO-520 (100 nM), TAK960 (100 nM) and NMS (500 nM) for 72 h. Expression was normalised to 5S rRNA. n = 3. (**C**) qRT-PCR analysis of pri-miRs levels in HEK293T cells treated with PLK1 inhibitors as indicated for 72 h. Expression was normalised to L19. (**A**–**C**) *Columns:* mean ± SEM for a minimum of three independent experiments. (**D**) Western blot analysis of phospho-TCTP (Ser46), phospho-Histone H3 (Ser10) and cyclin E1 protein levels in HEK293T cells treated with DMSO, RO-520 (100 nM) or NMS (500 nM) for 5, 24 or 72 h (**i**). β-tubulin was used as a control for loading. (**ii**–**iv**) Densitometry was performed using ImageJ (version 1.53t) and protein levels shown relative to β-tubulin. * *p <* 0.05*,* # *p* < 0.001, and ‡ *p* < 0.0001. Independent biological repeats are shown in Figure S8. See also Figures S6–S10.

Given the reduction in mature miR levels following PLK1 inhibition, we hypothesised that PLK1 may modulate pri-miR-to-pre-miR processing at the level of the Drosha Microprocessor through phosphorylation, consistent with previous reports of kinase-regulated Drosha activity [13]. In these circumstances, pri-miR levels would be decreased by PLK1 overexpression and increased by PLK1 inhibition or silencing, whilst pre- and mature miR levels would show the opposite, i.e., be increased by PLK1 overexpression and decreased by PLK1 inhibition. Indeed, both WT and constitutively active PLK1 significantly reduced pri-miR levels (Figure 2A) with host genes for intronic miRs unaffected or marginally raised (Figure S6), and reduced levels of pre-miRs were observed upon PLK1 inhibition (stem-loop-specific primers are used to amplify pre-miRs from cDNA prepared from size-selected RNA < 200 nt, other pre-miRs assayed were undetectable, presumably due to their rapid turnover—Figure 2B). However, qRT-PCR showed a substantial *decrease* in levels of all pri-miRs assessed when HEK293T cells were treated with a panel of PLK1 inhibitors (Figure 2C). PLK1 is a key mediator of cell cycle progression that facilitates the G_2_/M checkpoint and mitotic execution. Thus, decreased pri-miR levels upon PLK1 inhibitor treatment were hypothesised to be due to repressed transcription observed during M-phase arrest induced by PLK1 inhibition, particularly since levels of housekeeping transcripts were also reduced under these conditions, albeit to a lesser extent (Figure S7)**,** likely reflective of differing transcript half-lives. The activity of several luciferase-based Drosha Microprocessor reporters (in which luciferase activity is inversely proportional to Drosha activity) was increased by PLK1 inhibitor treatment. Whilst consistent with reduced Drosha activity under these conditions (Figure S8), these effects may also be attributable to reduced transcription in M-phase arrested cells, as discussed above.

Indeed, significant M-phase accumulation of both HEK293T and MCF7 cells was observed 24 h and 72 h post PLK1 inhibitor treatment, but not at 5 h, as assessed by Western blotting for the M-phase marker, phospho-Histone H3 (Ser^10^) (Figure 2D and Figure S9). Successful PLK1 inhibition was confirmed via phospho-TCTP (Ser^46^) Western blotting (Figure 2D and Figure S9). This data confirm that PLK1 modulation does indeed modulate cell cycle progression, and that transcriptional shut-down following PLK1 inhibition may be responsible for effects on pri-, pre- and mature miR levels at 72 h. In addition, it is possible that reduced proliferation rates resulting from M-phase stalling (Figure S10) may also impact miR biogenesis, although effects on mature miRs are observed at 5 h, prior to any PLK1 inhibitor-associated reduction in cell proliferation (see below).

**Figure 3:**
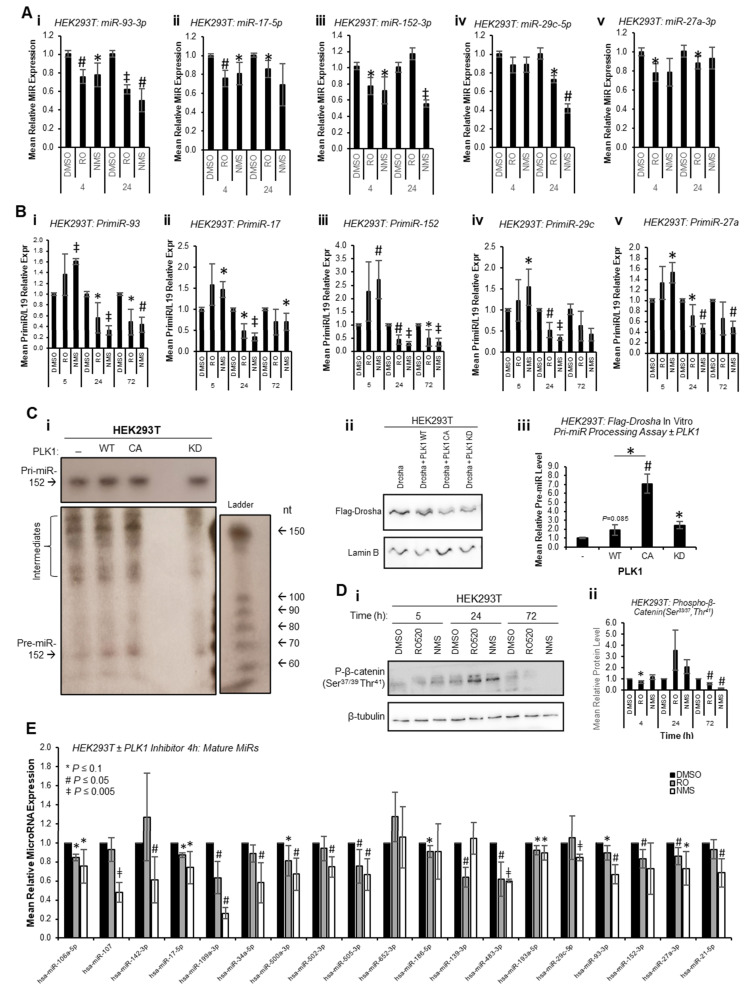
**PLK1 modulates microRNA biogenesis independently of its effects of cell cycle progression**. (**A**,**B**) qRT-PCR analysis of (**A**) RNA-seq-identified PLK1-regulated miRs (**i**—miR-93-3p, **ii**—miR-17-5p, **iii**—miR-152-3p, **iv**—miR-29c-5p, **v**—miR-27a-3p) and (**B**) pri-miRs (**i**—primiR-93, **ii**—primiR-17, **iii**—primiR-152, **iv**—primiR-29c, **v**—primiR-27a) in HEK293T cells treated with treated with DMSO, RO-520 (100 nM) or NMS (500 nM) for 4 and 24 h (**A**) or 4, 24 or 72 h (**B**). Data were normalised to U6 (**A**) and L19 (**B**)*. Columns:* mean ± SEM for a minimum of three independent experiments. (**C**) In vitro pri-miR processing assay analysis of Drosha activity in response to PLK1 inhibition. In vitro-transcribed, ^32^P radio-labelled artificial pri-miR-152 was incubated with Flag-Drosha immunoprecipitates from HEK293T cells transfected with Flag-Drosha ± WT, constitutively active (T^210^D) or kinase-dead (K^82^R) PLK1 for 48 h (**i**). n = 3. Independent biological repeats are shown in Figure S10. Pre-miR bands were quantified corrected for Flag-Drosha input levels (see **ii**,**iii**). (**ii**) Western blot analysis of Drosha protein levels in lysates of HEK293T transfected with Flag-Drosha ± WT, constitutively active (T^210^D) or kinase-dead (K^82^R) PLK1 for 48 h and used as input for in vitro pri-miR processing assays. Lamin B was used as a control for loading. (**iii**) Densitometry was performed using ImageJ and pre-miR levels are shown relative to Lamin B-normalised Flag-Drosha input protein levels ± SEM. (**D**) Western blot analysis of phospho-β-catenin (Ser^37/39^ Thr^41^) protein levels in HEK293T cells treated with DMSO, RO-520 (100 nM) or NMS (500 nM) for 4, 24 and 72 h (**i**). β-tubulin was used as a control for loading. Densitometry was performed using ImageJ and protein levels shown relative to β-tubulin (n = 3) (**ii**). Independent biological repeats are shown in Figure S12**.** (**E**) miR qPCR array analysis of HEK293T cells treated with DMSO, RO-520 (100 nM) or NMS (500 nM) for 4 h (n = 4). MiR levels were normalised to UniSp6 spike-in. Note that Ct values of assayed miRs ranged from 19 to 29, and for pri-miRs, from 15 to 33. See also Figures S11–S14. * *p <* 0.05*,* # *p* < 0.001, and ‡ *p* < 0.0001.

To circumvent the impact of PLK1 inhibition on transcription due to cell cycle arrest at M-phase, and to assess whether PLK1 can alter miR biogenesis prior to inhibitor-induced cell cycle changes, mature miR levels were assessed at 4 h and 24 h post inhibitor treatment. Mature miR levels were reduced at both time points across all identified PLK1-regulated miRs (Figure 3A). Further, pri-miR levels were increased 4 h post PLK1 inhibitor treatment and decreased at 24 and 72 h (Figure 3B). Together, these data suggest that inhibition of PLK1 represses pri-miR-to-pre-miR processing during miR biogenesis. In additional support of this, in vitro pri-miR processing assay, in which in vitro-transcribed and radiolabelled pri-miR-152 was incubated with immunoprecipitated Flag-Drosha ± WT, constitutively active or kinase-dead PLK1 revealed increased pre-miR levels in the presence of WT and constitutively active PLK1, and reduced levels with kinase-dead PLK1 (Figure 3C and Figure S11). Similar results were obtained using a ‘universal’ consensus pri-miR (although some non-specific, background cleavage was evident (processed products were obtained following incubation of in vitro-transcribed pri-miR with Flag beads incubated with cells *not* containing Flag-Drosha), a band consistent with the expected size of the pre-miR liberated from the consensus pri-miR (64 nt) was observed in Flag-Drosha lanes, and showed decreased intensity for lanes in which cells were treated with PLK1 inhibitors) (Figure S12). All of the above affects were assumed to be attributable to PLK1, since PLK1 inhibitors did not profoundly affect activity of GSK3β as assessed by phospho-β-catenin (Ser^37/39^,Thr^41^—Figure 3D, Figures S13 and S14), another kinase known to modulate Drosha activity [13].

To provide evidence as to whether early cell cycle-independent PLK1 effects of miR biogenesis are a universal event or restricted to a small sub-set of mature miRs, miR qPCR arrays were performed for HEK293T cells treated with RO or NMS for 4 h to assess levels of 30 miRs not previously described as PLK1-regulated (confirmed PLK1i-modulated miR-93-3p and miR-152-3p were included as positive controls). Of the 19 miRs consistently expressed across all four biological repeats, it was shown that 14 were significantly downregulated upon 4 h PLK1i, with an additional 3 trending towards significance (*p* ≤ 0.1) and 1 unchanged (miR-652-3p) (Figure 3E). This suggests that the majority of miRs are regulated by PLK1 independently of its effects on cell cycle progression. Regulation of these miRs is likely not observed at 72 h in the majority of cases (RNA-seq, Figure 1) due to consequences of wide-spread transcriptional shut-down during PLK1 inhibitor-induced M-phase arrest. Together, the above data support the hypothesis that PLK1 promotes Drosha-mediated pri-miR-to-pre-miR processing of a large subset of miRs independently of its cell cycle-modulatory roles.

**Figure 4:**
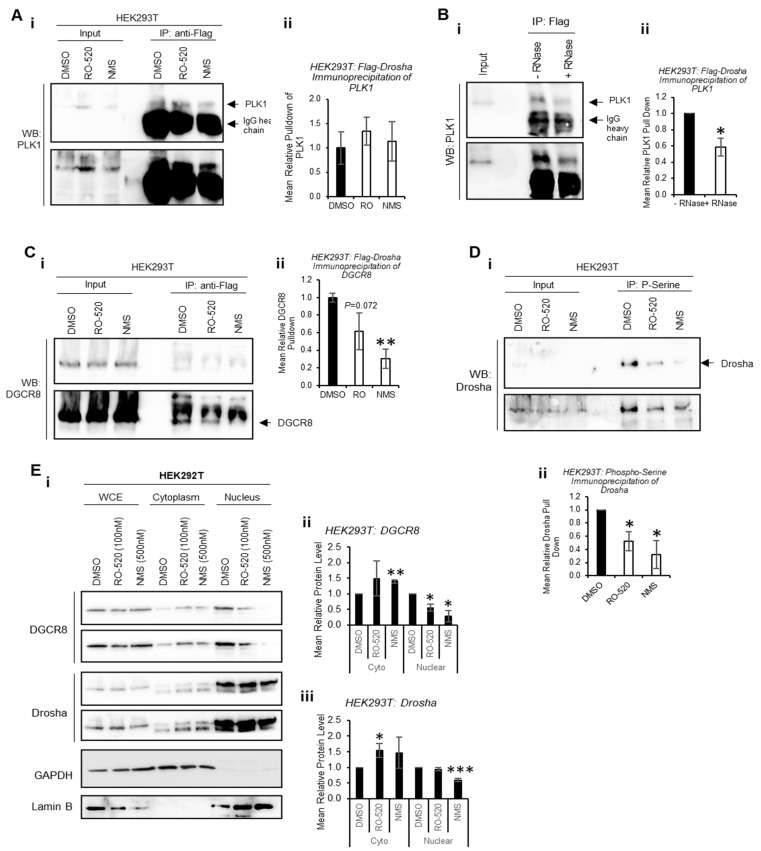
**PLK1 associates with Drosha in an RNA-dependent manner to modulate Drosha:DGCR8 interaction through its kinase activity**. (**A**–**C**) Western blot analysis of (**A**,**B**) PLK1 and (**C**) DGCR8 protein levels in Flag immunoprecipitates of HEK293T cells transfected with Flag-Drosha and treated with DMSO, RO-520 (100 nM) or NMS (500 nM) for 16 h (**A**(**i**),**C**(**i**)), or with RNase A (**B**(**i**)). Additional biological replicates are shown in Figure S14. Bands were quantified using ImageJ (**A**(**ii**),**B**(**ii**),**C**(**ii**)). (**D**) Western blot analysis of Drosha protein levels in phospho-serine immunoprecipitates of HEK293T cells treated with DMSO, RO-520 (100 nM) or NMS (500 nM) for 16 h. Additional biological replicates are shown in Figure S15. (**E**) Western blot analysis of DGCR8 and Drosha protein levels in whole-cell extracts (WCEs), cytoplasmic and nuclear fractions of HEK293T cells treated with DMSO, RO-520 (100 nM) or NMS (500 nM) for 3 h (**i**). GAPDH and Lamin B were used as cytoplasmic and nuclear controls, respectively. Additional biological replicates are shown in Figure S16. (**ii**,**iii**) Densitometry was performed using ImageJ and protein levels shown relative to WCE levels normalised to appropriate loading control. *Columns:* mean ± SEM for three independent experiments. * *p <* 0.05, ** p < 0.001 *** *p* < 0.0001. Throughout, two exposures of the same blot are shown, for clarity. See also Figures S15–S17.

To determine whether PLK1 interacts directly with Drosha to modulate its activity, co-immunoprecipitation assays were performed. Flag-Drosha interaction with endogenous PLK1 was confirmed, and this was not significantly altered by PLK1 inhibition (Figure 4A and Figure S15A). Interestingly, PLK1 levels in whole-cell lysates (input) were increased by PLK1 inhibition, perhaps as a feedback response to reduced PLK1 activity. To assess whether PLK1 interaction with Drosha required the presence of RNA, co-immunoprecipitation of PLK1 with Flag-Drosha was performed ± RNase. RNAse treatment reduced PLK1 association with Drosha when RNAs were degraded (Figure 4B and Figure S15B), suggesting enhanced association between PLK1 and Drosha in the presence of RNA (likely pri-miRs). Immunoprecipitation was also used to assess the effects of PLK1 inhibition on the interaction between Drosha and its key cofactor, DGCR8. Both PLK1 inhibitors reduced Drosha association with DGCR8, which may in part explain reduced Drosha activity upon PLK1 inhibition (Figure 4C and Figure S15C).

Drosha is known to be phosphorylated by a number of kinases to modulate its activity and localization [13,29,30]. To provide evidence for specific PLK1 phosphorylation of Drosha, HEK293T cells were treated ± PLK1 inhibitors (16 h) and immunoprecipitation performed using anti-phospho-serine agarose beads. This showed that PLK1 inhibition reduces levels of serine-phosphorylated Drosha, consistent with direct PLK1 phosphorylation of Drosha (Figure 4D and Figure S16). As Drosha phosphorylation has been shown to alter its localisation, we assessed the impact of PLK1 inhibition on Drosha and DGCR8 subcellular localisation via cell fractionation. We selected 3 h treatment to avoid significant PLK1i-induced M-phase arrest, when nuclear membrane breaks down. Nuclear protein levels of Drosha and DGCR8 were reduced upon PLK1 inhibition, with concomitant increases in cytoplasmic levels of both proteins (Figure 4E and Figure S17). This suggests that inhibition of PLK1 kinase activity impacts localisation of key Microprocessor components.

**Figure 5:**
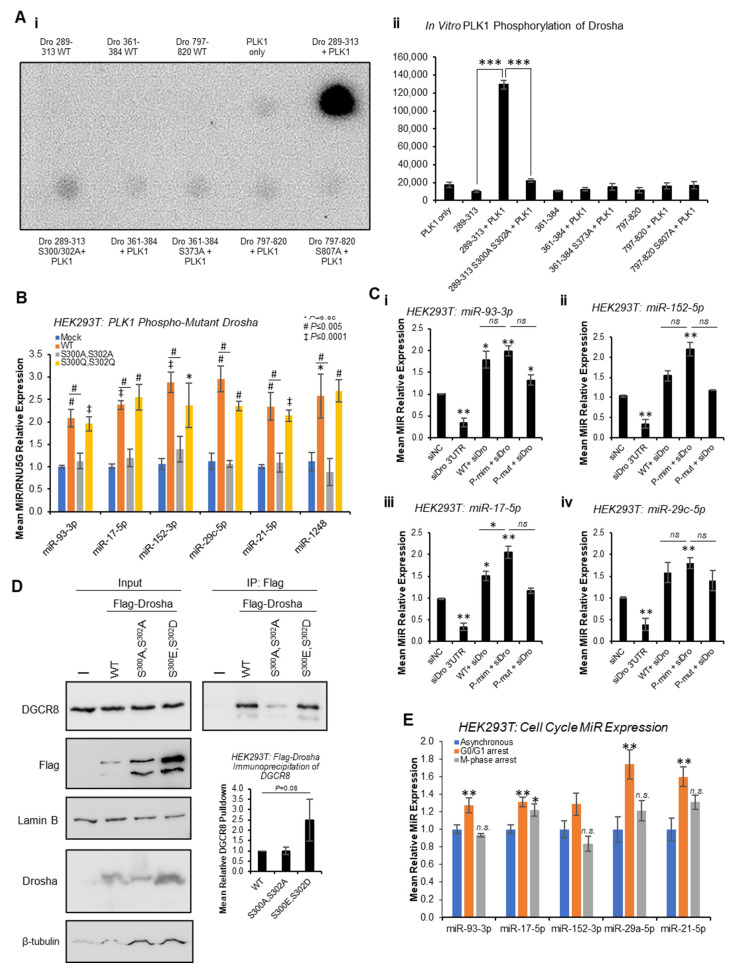
**PLK1 phosphorylates Drosha at S^300^ and/or S^302^ but not S^373^ and S^807^ to enhance Drosha activity independently of its cell cycle-modulating effects**. (**A**) In vitro kinase assay analysis of PLK1 phosphorylation of WT and phospho-site-mutant Drosha peptides (**i**). Indicated Drosha peptides were incubated with recombinant PLK1 in the presence of α-^32^P-ATP. Quantification was performed using ImageJ, n = 4. Additional biological replicates are shown in Figure S18. (**B**) qRT-PCR analysis of mature miR levels in HEK293T cells transfected with WT, phospho-mutant (S^300^A,S^302^A) or phospho-mimic (S^300^E,S^302^D) Flag-Drosha for 72 h. Expression was normalised to RNU5G, n = 3. (**C**) qRT-PCR analysis of mature miR levels in HEK293T cells transfected with Drosha 3′UTR siRNA and siRNA-resistant Flag-Drosha WT, phospho-mimic (S^300^E,S^302^D) or phospho-mutant (S^300^A,S^302^A) for 72 h, n = 3 Expression was normalised to RNU5G. **i**—miR-93-3p, **ii**—miR-152-3p, **iii**—miR-17-5p, **iv**—miR-29c-5p. (**D**) Western blot analysis of DGCR8 protein levels in Flag immunoprecipitates of HEK293T cells transfected with WT, phospho-mutant (S^300^A,S^302^A) or phospho-mimic (S^300^E,S^302^D) for 72 h. Quantification was performed using ImageJ and immunoprecipitated DGCR8 normalised to loading control-corrected Drosha protein levels in inputs. Additional biological replicates are shown in Figures S21 and S22, n = 3. *Columns:* mean relative DGCR8 protein levels normalised to β-tubulin-corrected Drosha input protein levels ± SEM for three independent experiments. (**E**) qRT-PCR analysis of mature miR levels in asynchronous, G_0_/G_1_-arrested and M-phase-arrested HEK293T. Expression was normalised to 5S rRNA, n = 3. *Columns:* mean ± SEM, *n.s.*—non-significant, * *p* < 0.05, ** *p* < 0.001, *** *p* < 0.0001. Confirmation of cell cycle arrest is shown in Figure S23. See also Figures S18–S23.

Quantitative phospho-proteomics has previously identified putative PLK1 phosphorylation sites at S^300^ and S^807^ of Drosha [29]. In addition, PhosphoSitePlus^®^ predicts a potential PLK1 phosphorylation site at S^373^, and we previously showed Drosha to be phosphorylated by GSK3β at S^300^ and S^302^ [13]. To assess whether PLK1 can phosphorylate Drosha at these sites in vitro, in vitro kinase assays were performed whereby Drosha peptides spanning these putative phosphorylation sites were incubated with recombinant PLK1 in the presence of ^32^P-ATP. This showed strong phosphorylation of Drosha 289-313 that was almost entirely abrogated when S^300^ and S^302^ were mutated to alanine (Figure 5A and Figure S18). Minimal phosphorylation of other Drosha peptides (361-384 and 797-820) was observed, and this was not altered by putative phosphorylation site mutation (Figure 5A and Figure S18). These data support PLK1 phosphorylation of Drosha at S^300^ and/or S^302^, but not S^373^ or S^807^.

To confirm the impact of PLK1 phosphorylation of Drosha at S^300^/S^302^, qRT-PCR of previously identified PLK-regulated miRs was performed following transfection of HEK293T cells with WT, phospho-mutant (S^300^A,S^302^A) or phospho-mimic (S^300^E,S^302^D) Drosha. Whilst WT Drosha significantly increased levels of all mature miRs assayed compared to mock-transfected cells, S^300^A,S^302^A phospho-mutant Drosha significantly reduced mature miRs compared to WT (Figure 5B). S^300^E,S^302^D phospho-mimic Drosha did not significantly increase levels of PLK1-regulated miRs compared to WT, possibly due to maximal miR synthesis in the presence of exogenous WT Drosha (Figure 5B). Pri-miR levels were generally non-significantly higher in the presence of S^300^A,S^302^A-mutant Drosha, as compared to WT (Figure S19). To more accurately delineate the effects of phospho-mimic and phospho-mutant Drosha, HEK293T cells were transfected with the above Drosha mutants following knockdown of endogenous Drosha using siRNA targeting Drosha 3′UTR (absent from mutant Drosha expression constructs). Similarly to Figure 5B, both WT and S^300^E,S^302^D phospho-mimic Drosha significantly increased mature miRs levels, with a greater effect observed for the phospho-mimic (Figure 5C and Figure S20). S^300^A,S^302^A phospho-mutant Drosha did not significantly alter mature miR levels, with the exception of miR-93-3p, where a significant increase was observed (Figure 5C). This suggests that phosphorylation at residues other than S^300^ and S^302^ may impact Drosha activity. Alternatively, this may be reflective of the lower expression of phospho-mutant Drosha versus phospho-mimic and WT Drosha (Figure S21).

IP analysis also showed 2.5-fold increased phospho-mimic Drosha interaction with DGCR8 as compared to WT (Figure 5D and Figure S21), albeit non-significant, suggesting that PLK1 phosphorylation of Drosha enhances its interaction with microprocessor cofactors. S^300^A,S^302^A phospho-mutant Drosha did not show altered association with DGCR8 compared to WT, possibly suggesting that phosphorylation events at other sites and/or additional post-translational modifications may contribute to Drosha:DGCR8 interactions (Figure 5D, Figures S21 and S22).

Since PLK1 inhibition significantly modulated Drosha localisation (Figure 4E), and Drosha phosphorylation is known to alter its localization [13], we assessed localisation of phospho-mutant and -mimic Drosha in comparison to WT protein. Notably, S^300^A,S^302^A and S^300^E,S^302^D Flag-Drosha did not show significantly altered subcellular localisation as compared to WT Flag-Drosha, with the exception that phospho-mutant Drosha showed slightly but significantly reduced cytoplasmic localisation as a proportion of total cellular protein (Figure S23). Therefore, we conclude that PLK1-induced phosphorylation of Drosha at S^300^ and S^302^ alters Drosha activity, but has little effect on localisation. We suggest that other PLK1-phosphorylated Drosha residues may be responsible for the effects of PLK1 inhibition on Drosha Microprocessor localisation, or alternatively, that additional PLK1-regulated kinases may be responsible for localisation-altering phosphorylation of Drosha.

Finally, to confirm that effects of PLK1 inhibition on mature miR levels are not simply reflecting broader miR alterations due to PLK1i-induced stalling of cells at M-phase, qPCR for PLK1-modulated miRs was performed following serum starvation (to arrest cells in G_0_/G_1_) and nocodazole treatment (for M-phase arrest), respectively (Figure 5E and Figure S24). Whilst some PLK1 regulated miRs were found to be significantly increased during G_0_/G_1_ arrest, and miR-17-5p was also elevated during M-phase arrest, no miRs were significantly decreased during M-phase arrest (Figure 5E) in a manner similar to the observed decrease in miR levels following PLK1 inhibition (Figure 1). This, in combination with the effects of PLK1 inhibition on Drosha and DGCR8 subcellular localisation occurring prior to accumulation of cells in M-phase in response to PLK1 inhibition, suggests that PLK1 regulates miR biogenesis independently of its effects on cell cycle progression.

**Figure 6:**
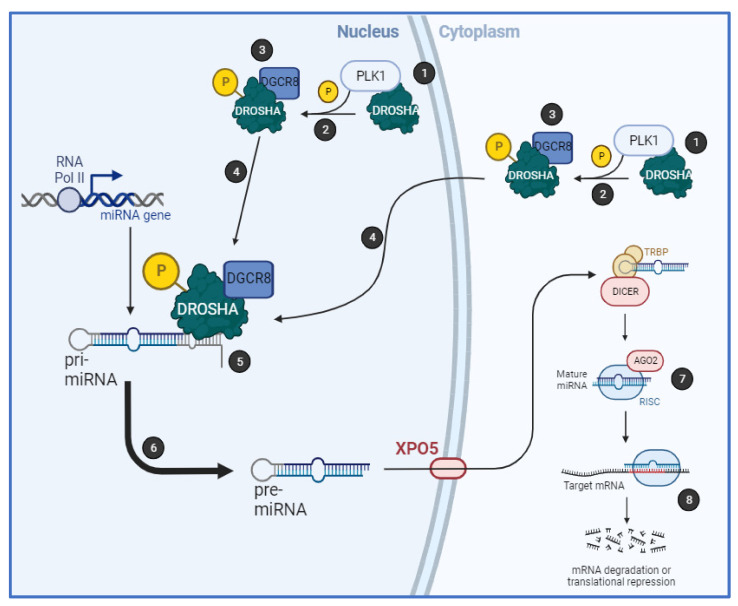
**Mechanism of PLK1 regulation of microRNA biogenesis**. (1) PLK1 associates with Drosha in an RNA-dependent manner (most likely in the nucleus but also possibly in the cytoplasm) leading to (2) its phosphorylation of Drosha at S^300^ and/or S^302^. This leads to (3) increased Drosha:DGCR8 association, (4) increased Drosha and DGCR8 nuclear localisation/retention, and (5) enhanced pri-miR association. This increases pri-miR-to-pre-miR processing (6), resulting in increased mature levels of PLK1-regulated miRs and downstream target regulation (7,8).

Together, we have shown that PLK1 regulates biogenesis of a subset of miRs by binding to the key microprocessor component, Drosha, in an RNA-dependent manner, resulting in its phosphorylation at S^300^ and/or S^302^ and increased cleavage of pri-miRs. PLK1 activity increases Drosha nuclear localisation and association with cofactor, DGCR8 (Figure 6). Importantly, PLK1 modulation of miR biogenesis is independent of its cell cycle-regulatory activity.

## 3. Discussion

MiR biogenesis is a complex, multi-step process regulated by a diverse array of signalling pathways. Such regulation serves to control miR levels both in a spatial and temporal manner. Modulated expression of miR subsets by various cofactors and binding partners permits subtle regulation of signalling pathway outputs and transcriptional programmes. This impacts almost all biological processes, from development to cardiac function. Perturbation of such regulatory networks can contribute to disease pathogenesis, as has been particularly well described in cancer, where reduced processing contributes to widespread miR downregulation [11,12]. A number of kinases have been previously identified as phosphorylating components of the key miR-processing complexes, the Drosha-containing Microprocessor and the Dicer complex, with some shown to impact miR biogenesis as described in the Introduction. It is likely that conformational changes induced by phosphorylation alter interactions between key RNases (Drosha and Dicer) and their cofactors, extent of substrate association, substrate specificity or protein localisation, permitting such factors to rapidly and subtly alter miR synthesis. Previous work from our laboratory identified GSK3β as a key modulator of global miR biogenesis as a Microprocessor component: GSK3β binds to p72 and DGCR8 in the microprocessor in an RNA-dependent manner, leading it to phosphorylate Drosha at S^300^ and S^302^. This increases Drosha association with cofactors and pri-miRs, and enhances its RNase and pri-miR cleavage activity, increasing mature miR levels [13].

Given the frequent dysregulation of kinases in cancer, and the clinical approval of a number of kinase inhibitors for treatment of a broad spectrum of diseases, it is notable that the contribution of the kinome of miR regulation has not been thoroughly investigated. Indeed, identification of kinase-regulated miR signatures may serve as a source of informative treatment response-predictive biomarkers. Kinase-regulated miRs may also themselves represent therapeutic targets. To identify miRs dysregulated by PLK1, small RNA-seq was performed on HEK293T cells treated with two highly specific PLK1 inhibitors for 72 h. Ten shared regulated miRs were identified and validated via qPCR in response to additional PLK1 inhibitors and PLK1 siRNA. Further, these identified PLK1 inhibitor-downregulated miRs were significantly upregulated in cells transfected with WT PLK1, and to a greater extent, in cells transfected with constitutively active PLK1. This supports the hypothesis that PLK1 enhances biogenesis of a miR subset. Importantly, the PLK inhibitor-regulated miRs showed strong nucleotide biases within the seed region. This may suggest that PLK1-modulated miRs could converge upon targets containing miR binding sites with high sequence similarity, potentially regulating multiple members of the same protein family, with functions in the same biological processes. Indeed, common pathways shared by validated PLK1-regulated miR targets include NOTCH and Akt signalling, p53 activity and senescence, all of which are strongly implicated in tumourigenesis. Notable shared targets (from miRTarBase database of validated miR targets) include those with roles in cell cycle progression (CDKN1A [miR-152-3p, miR-1306-5p, miR-1248]—negative regulator of G_1_ transition; CSKN2A1 [miR-152-3p, miR-1306-5p, miR-1248]—integration node for cell cycle and apoptotic pathways; WASL [miR-152-3p, miR-1248]—involved in actin polymerisation during mitosis and cytokinesis; PDS5A [miR-1306-5p, miR-93-3p]—regulates sister chromatid cohesion during mitosis), and immune response (CXCL5 [miR-1248, miR-93-3p]—pro-tumourigenic cytokine; CSKN2A1—response to viral infection; CRCP [miR-1306-5p, miR-93-3p]—sensor of non-self DNA and activator of innate immunity). The regulation of these factors by PLK1 and its downstream miRs should be further investigated given the current advancement of PLK1 inhibitors through cancer clinical trials.

Given that previous studies have identified mechanistically undescribed PLK1 phosphorylation sites on Drosha, we hypothesised that the PLK1 regulation of miR biogenesis may occur at the pri-miR-to-pre-miR processing step. To investigate this, we examined the effect of PLK1 inhibitor treatment on pri-miR levels. After 72 h, significant and extensive decreases in all assayed pri-miRs were observed, contrary to the expected accumulation of pri-miRs following PLK1 inhibition. However, this is likely attributable to repressed transcription in M-phase arrested cells, since PLK1 is a key mediator of cell cycle progression that facilitates the G_2_/M checkpoint. A key question was therefore whether PLK1 regulates miR biogenesis independently of its effects on cell cycle progression. Three pieces of evidence were obtained in support of cell cycle-independent PLK1-promoted miR biogenesis: (i) mature miR levels were significantly decreased and pri-miR levels significantly increased after 4 h PLK1 inhibitor treatment (this time being insufficient to induce significant cell cycle arrest at G_2_/M), (ii) in vitro pri-miR processing assays showed a significant increase in pre-miR levels following WT or constitutively active PLK1 transfection of HEK293T cells, and iii) pri-miR levels were significantly decreased following WT or constitutively active PLK1 overexpression. Together these data support the hypothesis that PLK1 promotes Drosha-mediated pri-miR-to-pre-miR processing. This is consistent with our prior demonstration that GSK3β increases Drosha cleavage of pri-miRs and suggests that several kinases may converge upon Drosha to regulate distinct miR subsets. To ascertain whether cell cycle-independent PLK1 promotion of miR biogenesis is a miRome-wide event, or restricted to a small miR subset, miR qPCR arrays were performed for HEK293T cells treated with PLK1 inhibitors for 4 h. It was shown that 74% of detectable assayed miRs were significantly downregulated upon PLK1 inhibition, with three trending downwards and one unchanged (miR-652-3p). This suggests that the majority of miRs are regulated by PLK1 in a cell cycle-independent manner.

To further detail the mechanism by which PLK1 modulates Drosha activity, we first assessed the ability of PLK1 to interact with Drosha (note that PLK1 is found in both cytoplasm and nucleus—proteinatlas.org, accessed on 11 June 2021). PLK1 was found to associate with Drosha, but this interaction was not altered by PLK1 inhibition. Notably, whole-cell PLK1 protein levels are increased following PLK1 inhibition, possibly as a feedback response to pharmacological inhibition. It was, however, observed that Drosha:PLK1 interaction is reduced by RNase treatment, suggesting that presence of pri-miRs is required for optimal association between Drosha and PLK1. One mechanism by which PLK1 may increase Drosha activity or alter its substrate specificity could be through altering its interactions with key cofactor, DGCR8. Indeed, it was confirmed that DGCR8 protein levels in Flag-Drosha immunoprecipitates are reduced following PLK1 inhibition, confirming that PLK1 enhanced Drosha:DGCR8 interaction. We also demonstrated that PLK1 kinase activity is involved in modulating Drosha activity, since levels of serine phosphorylated Drosha are reduced following PLK1 inhibition.

In our previous work, we showed that GSK3β phosphorylates Drosha at S^300^ and S^302^ to increase its nuclear localisation. For this reason, we wondered whether pharmacological inhibition of PLK1 kinase activity might also impact its nuclear localisation. Indeed, we found that treatment with PLK1 inhibitors for 3 h (insufficient to cause M-phase arrest and nuclear membrane breakdown) resulted in a significant increase in cytoplasmic levels of both Drosha and DGCR8. Conversely, nuclear levels of both proteins were reduced. This suggests that PLK1 phosphorylation of Drosha can increase its nuclear localisation to promote pri-miR-to-pre-miR processing. It remains to be determined whether DGCR8 is also subject to PLK1 phosphorylation.

To establish which putative phospho-sites on Drosha are phosphorylated by PLK1, in vitro kinase assays were performed using Drosha peptides spanning predicted or validated Drosha phosphorylation sites. PLK1 was able to phosphorylate serine residues within the 289–313aa Drosha peptide, containing the S^300^ and S^302^ residues that we have previously shown to be phosphorylated by GSK3β. In confirmation of this, phospho-mutant Drosha S^300^A,S^302^A reduces mature levels of PLK1-regulated miRs as compared to WT Drosha, whilst phospho-mimic Drosha S^300^E,S^302^D increases mature miRs. Importantly, the impact of PLK1 on Drosha phosphorylation and localisation is independent of PLK1’s effects on cell cycle, since identified PLK1-regulated miRs are not altered in M-phase-arrested HEK293T cells, as compared to asynchronous cells. Further, phosphorylation at S^300^ and/or S^302^ has functional impacts on miR biogenesis, since Drosha S^300^A,S^302^A shows reduced association with DGCR8, and Drosha S^300^E,S^302^D shows increased association, compared to WT protein. Notably, although PLK1 inhibition altered Drosha and DGCR8 localisation, S^300^A,S^302^A and S^300^E,S^302^D phospho-mutant and -mimic Flag-Drosha did not show significantly altered subcellular localisation as compared to WT Flag-Drosha. We therefore conclude that PLK1-induced phosphorylation of Drosha at S^300^ and S^302^ alters Drosha activity, but not localisation. We suggest that other PLK1-phosphorylated Drosha residues may be responsible for effects of PLK1 inhibition on Drosha Microprocessor localisation, or alternatively, that additional PLK1-regulated kinases may be responsible for localisation-altering phosphorylation of Drosha.

Our findings agree with other recent data that provide evidence for kinase modulation of microprocessor components. PAK5 phosphorylates DDX5 at Thr^69^, leading to its enhanced sumoylation and stabilisation. This promotes DDX5/Drosha/DGCR8 complex formation and miR-10b processing in breast cancer [31]. Similarly, p38 MAPK phosphorylation of Drosha in primary cortical neurons resulted in its relocalisation from nucleus to cytoplasm, and a decrease in its protein levels in a rat transgenic model of Alzheimer’s disease [32]. Kinase regulation of miR biogenesis via altered microprocessor localisation appears to be conserved across species, since MPK3 was shown to phosphorylate the microprocessor component, HYL1 (hyponastic leaves 1) in *Arabadopsis thaliana* [33]. Like PLK1 phosphorylation of Drosha, this appears to be an important determinant of microprocessor subcellular localisation.

Together, our data show for the first time that PLK1 modulates biogenesis of a subset of miRs by phosphorylating Drosha at S^300^ and/or S^302^, resulting in increased Drosha association with DGCR8 and enhanced pri-miR-to-pre-miR processing, independently of PLK1 cell cycle regulatory activity. This raises the possibility that S^300^ and S^302^ serve as major integration points for signalling by a number of kinases, the relative activities of which will determine the relative biogenesis efficiency of different miR subsets. The identification of kinase-regulated miRs has implications for the use of miRs as kinase inhibitor response-predictive biomarkers in cancer and other diseases.

## 4. Materials and Methods

### 4.1. Cell Line Culture

HEK293T and MCF7 cells were cultured in DMEM supplemented with 10% foetal bovine serum with 2 mM L-glutamine at 37 °C with 5% CO_2_. Cell lines were tested for presence of mycoplasma on a monthly basis using MycoAlert Mycoplasma Detection Kit (Lonza, Basel, Switzerland). Annual cell line authentication was conducted using the MWG Eurofins Human Cell Line Authentications service.

### 4.2. RNA Isolation and Small RNA Enrichment

Total RNA was isolated from cells using the Monarch RNA Mini-Prep Kit (New England Biolabs, Ipswich, MA, USA). Small RNA fraction was prepared using the ‘Fractionation of Small and Large RNA’ protocol for the above kit.

### 4.3. Small RNA-Sequencing and Bioinformatics Analysis

QC, small RNA sequencing and bioinformatics analysis was conducted by Novogene (see Supplementary Methods). The nucleotide bias analysis shows the mean frequency of bases at each position for all known and novel miRs detected for DMSO condition, and for statistically significantly differentially expressed miRs vs. DMSO (RO420- and GSK461-treated samples). Mean of n = 3 samples per condition is shown. Mean numbers of detected miRs per treatment are 830 (DMSO), 770 (RO520) and 882 (GSK461).

### 4.4. Drug Treatments

PLK1 inhibitors were purchased from Selleckchem (Houston, TX, USA) and dissolved in DMSO. These were used to treat cells at final concentrations of 100 nM (GSK461361 [GSK461], TAK-960 and RO-5203280 [RO-520]) and 500 nM (NMS1286937 [NMS]). Equal volumes of vehicle (DMSO) were used as vehicle control.

### 4.5. Recombinant Proteins and Peptides

Recombinant human PLK1 was purchased from Bio-Techne (Minneapolis, MN, USA) (3804-KS-010). WT and phospho-site-mutant Drosha peptides corresponding to amino acids 289-313, 361-384 and 797-820 were synthesised by Sigma-Aldrich (St. Louis, MO, USA) and reconstituted in sterile TBS. Amino acid sequences were as follows:

Drosha 289-313 WT: RERHRHRDNRRSPSLERSYKKEYKR

Drosha 289-313 S300A,S302A: RERHRHRDNRRAPALERSYKKEYKR

Drosha 361-384 WT: KRARWEEEKDRWSDNQSSGKDKNY

Drosha 361-384 S373A: KRARWEEEKDRWADNQSSGKDKNY

Drosha 797-820 WT: YVKLRHLLANSPKVKQTDKQKLAQ

Drosha 797-820 S807A: YVKLRHLLANAPKVKQTDKQKLAQ.

### 4.6. Plasmids and Transfection

pCer-C3-PLK1, pCer-C3-PLK1(T210D) and pCer-C3-PLK1(K82R) were purchased from Addgene (Watertown, MA, USA) (#68132, 68133 and 68134, respectively). pcK-Flag-Drosha was a kind gift from V. Narry Kim (Seoul National University, Republic of Korea). JetPrime transfection reagent (Polyplus, Illkirch, France) was used to transfect above plasmids into cells according to the manufacturer’s protocol.

### 4.7. Western Blotting

Western blotting was performed as described [34]. Primary antibodies were rabbit anti-Drosha mAb (ab183732, Abcam, Cambridge, UK), mouse anti-PLK1 mAb (#4513, Cell Signalling, Danvers, MA, USA), mouse anti-Flag epitope tag M2 mAb (F1804, Sigma-Aldrich, St. Louis, MO, USA), rabbit anti-DGCR8 mAb (ab191875, Abcam), rabbit anti-GAPDH mAb (#2118, Cell Signalling), rabbit anti-Lamin B1 mAb (ab229025, Abcam), mouse anti-phospho-serine (P5747, Sigma-Aldrich) or mouse anti-β-actin mAb (ab6276, Abcam). Secondary antibodies were goat anti-mouse- or goat anti-rabbit-HRP as appropriate (12-349 and 12-348, respectively, Sigma-Aldrich). Detection was performed using Luminata Forte HRP substrate (Millipore, Burlington, MA, USA).

### 4.8. Pri-miR and Pre-miR qRT-PCR

Total RNA (500 ng) or small RNA fraction (<200 nt–100 ng) was reverse transcribed using RevertAid First Strand cDNA Synthesis kit (ThermoFisher Scientific, Altrincham, UK). cDNA was diluted 1:3–1:5 in nuclease-free H_2_O prior to qPCR using Fast SYBR Green Master Mix (ThermoFisher Scientific, Altrincham, UK) and primers at 250 nM. Primers were purchased from MWG Eurofins or Life Technologies. Sequences are shown in Supplementary Materials and Methods.

### 4.9. MiR-Specific qRT-PCR

An amount of 10 ng total RNA was used for miR-specific reverse transcription using the miRCURY LNA miRNA RT Kit (Qiagen, Hilden, Germany). cDNA was diluted 1:15–1:60 prior to qPCR using miRCURY LNA miRNA PCR assays and miRCURY LNA SYBR Green PCR Kit (both Qiagen).

### 4.10. RNA Immunoprecipitation

RNA immunoprecipitation was performed as described [13].

### 4.11. Co-Immunoprecipitation

Co-immunoprecipitation experiments were performed as described [13].

### 4.12. Subcellular Fractionation

Subcellular fractionation was performed as described [13].

### 4.13. In Vitro Kinase Assays

An amount of 400 ng recombinant PLK1 was incubated with 25 µg Drosha synthetic peptides, 50 µM cold ATP and 3 µL [γ-^32^P]-ATP (10 Ci/mmol 2 mCi/mL, PerkinElmer, Waltham, MA, USA) in kinase assay buffer (25 mM MOPS, 12.5 mM beta -glycerophosphate, 25 mM MgCl_2_, 5 mM EGTA, 2 mM EDTA, 0.25 mM DTT, pH7.2) for 1 h at 30 °C. Reactions were terminated via incubation at 65 °C for 20 min. Then, 10 µL of each reaction was dotted onto nitrocellulose membrane, left to dry for 2–3 min, washed ×3 with TBST and exposed to X-ray film.

### 4.14. Cell Synchronisation and Cell Cycle Arrest Induction

Nocodazole was used to arrest cells in M-phase: cells were serum-starved for 18 h to synchronise cells in G_0_/G_1_, followed by release into full medium in the presence of Nocodazole (50 ng/mL, 16 h). Flow cytometry was used to confirm M-phase arrest (see below).

### 4.15. Propidium Iodide Flow Cytometry Analysis of Cell Cycle Distribution

Cells fixed in 70% ethanol were pelleted by centrifugation, washed with PBS and resuspended in 500 µL FxCycle PI/RNase solution (ThermoFisher Scientific, Altrincham, UK) according to the manufacturer’s protocol and analysed on FACS Symphony, gating for live cells (FSC-A vs. SSC-A), single cells (YG610/20-H vs. YG610/20-A) and DNA content (YG610/20-A vs. count). Data were processed to calculate percentage of cells in Sub-G_1_. G_1_/G_0_, S and G_2_/M using Floreada (https://floreada.io/, accessed 3 April 2023).

### 4.16. Luciferase-Based Drosha Activity Reporter Assays

We previously generated a Drosha reporter in which CMV-driven transcription yields pri-miR-23a27a24-2 linked to the luc2p transcript. Drosha-mediated cleavage of pri-miR results in loss of luciferase activity, and hence, Drosha activity is inversely correlated with luminescence. This approach sensitively reported Drosha-mediated cleavage of specific pri-miR species in previous studies [13,35]. To investigate broader impacts of PLK1 across the miRnome, we generated an artificial universal pri-miR sequence based on a universal hairpin design sequence [36], incorporating all pri-miR features important for efficient Drosha processing [4,36,37,38,39]—‘STAND’ reporter. This was modified by site-directed mutagenesis for greater Drosha cleavage efficiency, to incorporate GCUG at 5′ Drosha cleavage site, GUCC at 3′ Drosha site.

Sequences of the artificial pri-miRs inserted into CMV-pGL4.18 between CMV promoter and luc2p are as follows: Standard (STAND)—CAAGAGAACAAAGTGGAGTCTTTGTTGCCCACACCCAGCTTCCCTGGCTCAAATTTGTGCCCATTCACATCTGTACATGAGACCGATGTGAATGCGCACAAATTAGAGCTTGGGAAGCATCTGCAGCAGAGCCTGCCTGGTGGCCCCTGAGAGATTTAAGCTT, Optimal (OPT): CAAGAGAACAAAGTGGAGTCTTTGTTGCCCACACCCAGCTTCCCTGGCGCTGATTTGTGCCCATTCACATCTGTACATGAGACCGATGTGAATGCGCACAAATTAGTCCTTGGGAAGCATCTGCAGCAGAGCCTGCCTGGTGGCCCCTGAGAGATTT, Pri-miR-21 (pri-21): TGTCGGGTAGCTTATCAGACTGATGTTGACTGTTGAATCTCATGGCAACACCAGTCGATGGGCTGTCTGACA.

The above reporter plasmids were stably transfected into MCF7 cells and monoclonal cell lines generated from G418-resistant single cells. Clonal cell lines were treated with PLK1 inhibitors for 96 h and luciferase assays performed using BrightGlo luciferase assay reagent (Promega, Madison, WI, USA) according to the manufacturer’s recommendations. Luciferase activity was corrected for PLK1 inhibitor effects on cell number by CellTiter Blue assay (Promega, Madison, WI, USA), performed according to the manufacturer’s instructions.

### 4.17. In Vitro Pri-miR Processing Assay

Artificial universal pri-miR sequence (OPT as above) or pri-miR-152 sequence was subcloned into pGemT in the Sp6 orientation. The plasmid was linearised using NCo1 (New England Biolabs, Ipswich, MA, USA) downstream of the pri-miR sequence and in vitro transcribed using the MEGAScript Sp6 in vitro transcription kit (ThermoFisher Scientific, Altrincham, UK) according to the manufacturer’s instructions in the presence of 4 μL α-32P-UTP (20 μCi/μL, 800 mCi/mmole, Perkin Elmer) and 0.5 mM cold UTP. Resultant RNA was isolated using phenol:chloroform, resuspended in RNA loading buffer (95% deionized formamide, 0.025% bromophenol blue, 0.025% xylene cyanol, 5 mM EDTA and 0.025% SDS), denatured by incubation at 95 °C for 5 min, and run on a 7% urea-polyacrylamide gel at 250 V in 0.5× TBE (pre-run for 1 h prior to loading). The gel was exposed to film and the radiolabelled transcript cut out. The gel slice was broken up using a pipette tip and incubated in 350 μL of RNA elution buffer (0.3 M sodium acetate pH 5.5, 2% SDS) overnight at 42 °C. Eluted RNA was precipitated with ice-cold ethanol and resuspended in 40 µL RNase-free TE buffer.

For isolation of Drosha Microprocessor, HEK293T cells were transfected with pCK-Flag-Drosha ± pCer-C3-PLK1, pCer-C3-PLK1(T210D) and pCer-C3-PLK1(K82R) using JetPrime (PolyPlus, Illkirch, France) according to the manufacturer’s protocol for 48 h. Transfected cells were then treated with 100 nM RO-520 or 500 nM NMS for 5 h as required. Cells were washed in ice-cold PBS, pelleted by centrifugation and resuspended in 500 μL lysis buffer (20 mM Tris-HCl (pH 8.0), 100 mM KCl and 0.2 mM EDTA plus protease and phosphatase inhibitors). Lysates were passed ×5 through a fine-gauge needle to aid lysis and incubated on ice for 10 min. Lysates were centrifuged at 13,000× *g* for 15 min at 4 °C and supernatant transferred to a fresh tube. Cleared lysates were incubated with pre-washed anti-FLAG antibody conjugated beads (Anti-FLAG M2 Affinity Gel, Sigma, A2220) resuspended in lysis buffer (20 µL original bead volume per sample) for 2 h at 4 °C, prior to washing x6 with lysis buffer. Drosha Microprocessor immobilised on FLAG beads was then used for in vitro pri-miR processing. Then, 15 uL immobilised Drosha Microprocessor was incubated with 3 μL of radio-labeled pri-miR, 0.75 μL RNase inhibitor, 8.25 μL RNase-free water and 3 μL 10× reaction buffer (64 mM MgCl_2_) for 90 min at 37 °C with occasional mixing. RNA was phenol:chloroform extracted, ethanol precipitated and resuspended in 15 μL RNA loading buffer as above. A 7% urea-polyacrylamide gel was prepared and pre-run at 250 V in 0.5× TBE for 60 min, during which radiolabelled RNA markers were prepared using the Decade™ Markers System (ThermoFisher Scientific, Altrincham, UK) according to the manufacturer’s instructions. Samples were heated to 95 °C alongside ladder for 5 min and then loaded directly onto the pre-run gel at 250 V in 0.5× TBE. The gel was exposed to Kodak X-AR5 autoradiography film in an intensifier screen overnight at −80 °C and developed. Expected in vitro-transcribed pri-miR size = 240 bp (artificial universal pri-miR) or 446 bp (pri-miR-152), and expected processed pre-miR size = 64 bp (artificial universal pre-miR) or 68 bp (pre-miR-152).

### 4.18. MiR qPCR Array Analysis

MiR qPCR arrays were performed using miRCURY LNA miRNA Custom PCR Panel (30 miRs) according to the manufacturer’s instructions. MiR assays included hsa-miR-100-5p, hsa-miR-106-5p, hsa-miR-107, hsa-miR-142-3p, hsa-miR-17-5p, hsa-miR-199a-3p, hsa-miR-299-5p, hsa-miR-34a-5p, hsa-miR-500a-3p, hsa-miR-502-3p, hsa-miR-505-3p, hsa-miR-652-3p, hsa-miR-155-5p, hsa-miR-186-5p, hsa-miR-143-5p, hsa-miR-139-3p, hsa-miR-147b-3p, hsa-miR-302b-3p, hsa-miR-34b-3p, hsa-miR-483-3p, hsa-miR-485-5p, hsa-miR-887-5p, hsa-miR-584-5p, hsa-miR-34c-5p, hsa-miR-193a-5p, hsa-miR-29c-5p, hsa-miR-93-3p, hsa-miR-152-3p, hsa-miR-27a-3p, hsa-miR-21-5p, UniSp6 (reverse transcription spike-in) and UniSp3 (qPCR spike-in).

### 4.19. MicroRNA Pathway Analysis

MiR pathway analysis was performed using MIENTURNET [40].

## Data Availability

The data presented in this study are available on request from the corresponding author.

## Supplementary Materials

The following supporting information can be downloaded at: https://www.mdpi.com/article/10.3390/ijms241814290/s1.
