# Supplementary material for: PLK1 Regulates MicroRNA Biogenesis through Drosha Phosphorylation
Source: Int J Mol Sci. 2023 Sep 19;24(18):14290. doi: 10.3390/ijms241814290 (PMC10531876; doi:10.3390/ijms241814290)
Supplement: Supplementary file 1 [file ijms-24-14290-s001.zip › PLK1 miR biogenesis paper supp figures and tables post-review 4.pdf]

GSK461 vs DMSO	log2FoldChange	padj
hsa-miR-1248	-3.145	7.23E-07
hsa-miR-511-5p	2.6332	1.46E-06
hsa-miR-891a-5p	3.2571	1.46E-06
hsa-miR-152-3p	1.5457	4.27E-06
hsa-miR-1306-5p	-1.8734	7.26E-05
hsa-miR-509-3-5p	2.0663	0.000198
hsa-miR-892a	2.0309	0.017393
hsa-miR-3688-3p	2.2838	0.021659
hsa-miR-3688-5p	2.2838	0.021659
hsa-miR-6716-3p	2.5435	0.021659
hsa-miR-139-3p	2.3904	0.025937
hsa-miR-93-3p	-1.2808	0.025937
hsa-miR-616-5p	-2.4012	0.029932
hsa-miR-483-5p	2.3626	0.031181
hsa-miR-2277-5p	-1.3697	0.034963
hsa-miR-29c-5p	-1.2142	0.039904

Table S1: Mature MicroRNAs Regulated by PLK1 Inhibitor, GSK461364 in HEK293T Cells. Padj = adjusted p value. Red = upregulated, blue = downregulated. Data relating to **Fig 1** are shown

RO520 vs DMSO	log2FoldChange	padj
hsa-miR-511-5p	2.9589	1.11E-07
hsa-miR-1306-5p	-2.4572	2.65E-05
hsa-miR-892a	2.8486	2.65E-05
hsa-miR-891a-5p	2.9103	0.000264
hsa-miR-152-3p	1.2413	0.00034
hsa-miR-378a-5p	-1.6829	0.000504
hsa-miR-4286	-2.5818	0.000504
hsa-miR-578	1.6541	0.001986
hsa-miR-183-5p	1.0586	0.002164
hsa-miR-509-3-5p	1.6569	0.002164
hsa-miR-219a-1-3p	1.7555	0.003948
hsa-miR-4661-5p	2.0139	0.004562
hsa-miR-99b-3p	1.5153	0.004872
hsa-miR-1248	-2.1529	0.005754
hsa-miR-193b-3p	-1.7357	0.00676
hsa-miR-212-5p	1.2614	0.006819
hsa-miR-2277-5p	-1.647	0.008204
hsa-miR-1296-5p	-1.8469	0.017606
hsa-miR-181a-2-3p	1.1131	0.017606
hsa-miR-23b-3p	-1.3383	0.017606
hsa-miR-365a-3p	-1.5879	0.017606
hsa-miR-486-3p	1.3083	0.017606
hsa-miR-500a-5p	-2.0656	0.017606
hsa-miR-1260b	-1.8747	0.018634
hsa-miR-30a-3p	1.2677	0.022904
hsa-miR-486-5p	1.305	0.022904
hsa-miR-331-3p	-1.4461	0.026473
hsa-miR-93-3p	-1.309	0.026473
hsa-miR-29c-5p	-1.0419	0.032357
hsa-miR-215-5p	1.626	0.03943
hsa-miR-93-5p	-1.6198	0.03943
hsa-miR-1288-3p	1.4389	0.048795
hsa-miR-17-5p	-1.7032	0.048795
hsa-miR-221-5p	-1.1293	0.048795
hsa-miR-98-5p	-1.1388	0.048795
hsa-miR-454-3p	-1.7582	0.049784

Table S2: Mature MicroRNAs Regulated by PLK1 Inhibitor, RO-5203280 in HEK293T Cells. Padj = adjusted p value. Red = upregulated, blue = downregulated. Data relating to **Fig 1** are shown

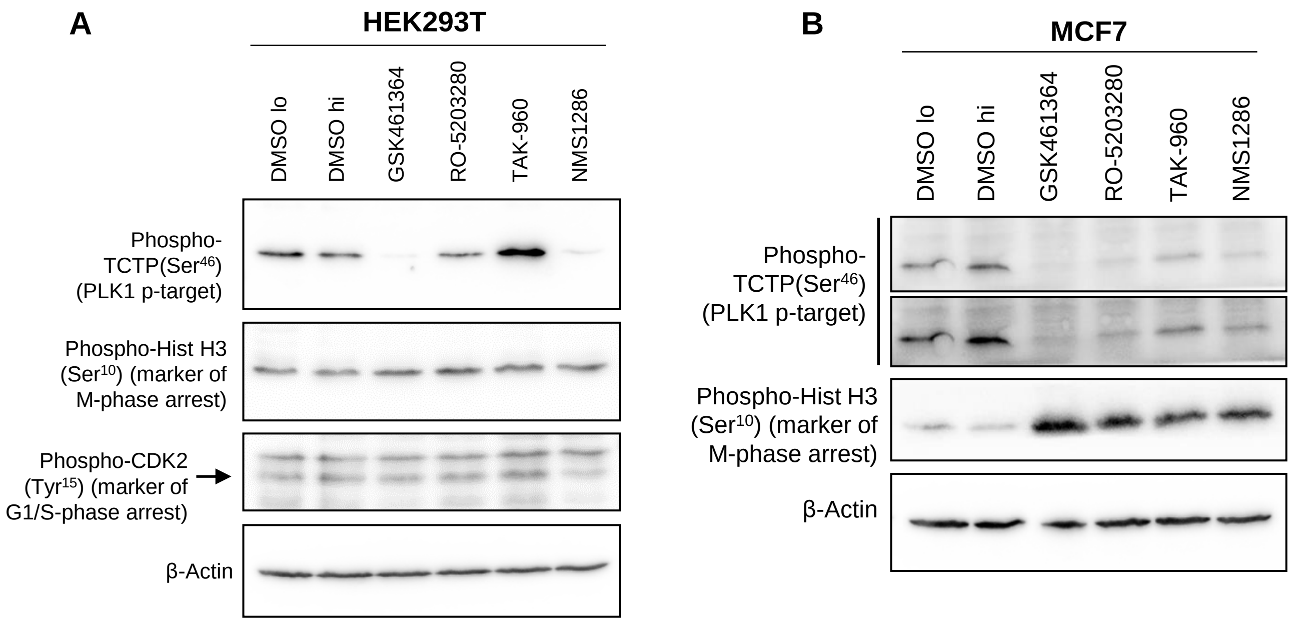

Figure S1: Treatment of HEK293T and MCF7 Cells with PLK1 Inhibitors Decreases Phosphorylation of PLK1 Target, TCTP, and Increases Phosphorylation of Histone H3 – a Marker of M-Phase Arrest. *A,B)* Western blot analysis of phospho-TCTP(Ser^{46}), phospho-Histone H3(Ser^{10}) and phospho-CDK2(Tyr^{15}) protein levels in *(A)* HEK293T and *(B)* MCF7 cells treated with DMSO, GSK461364 (100nM), RO-5203280 (100nM), TAK-960 (100nM) or NMS1286 (500nM) for 24h. β-actin was used as control for loading. N=1. Data relating to **Fig 1** are shown.

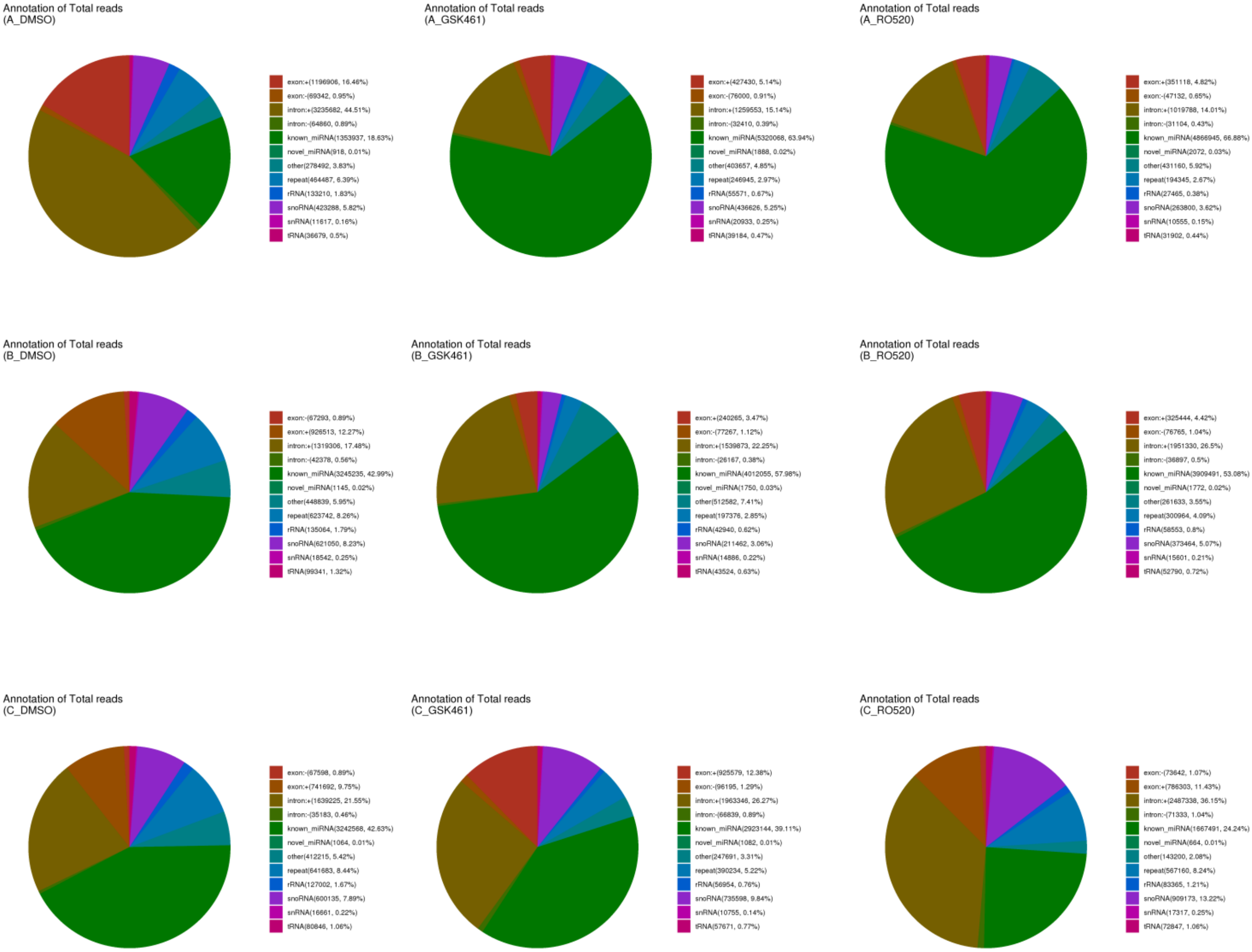

Figure S2: Percentages of Reads Mapping to Different Small RNA Types Following RNA-Seq of HEK293T Cells Treated with PLK1 Inhibitors. Pie charts show distribution of reads for different biological repeats (A, B and C), and in cells subject to treatment with DMSO, GSK461 and RO-520. Data relating to **Fig 1** are shown.

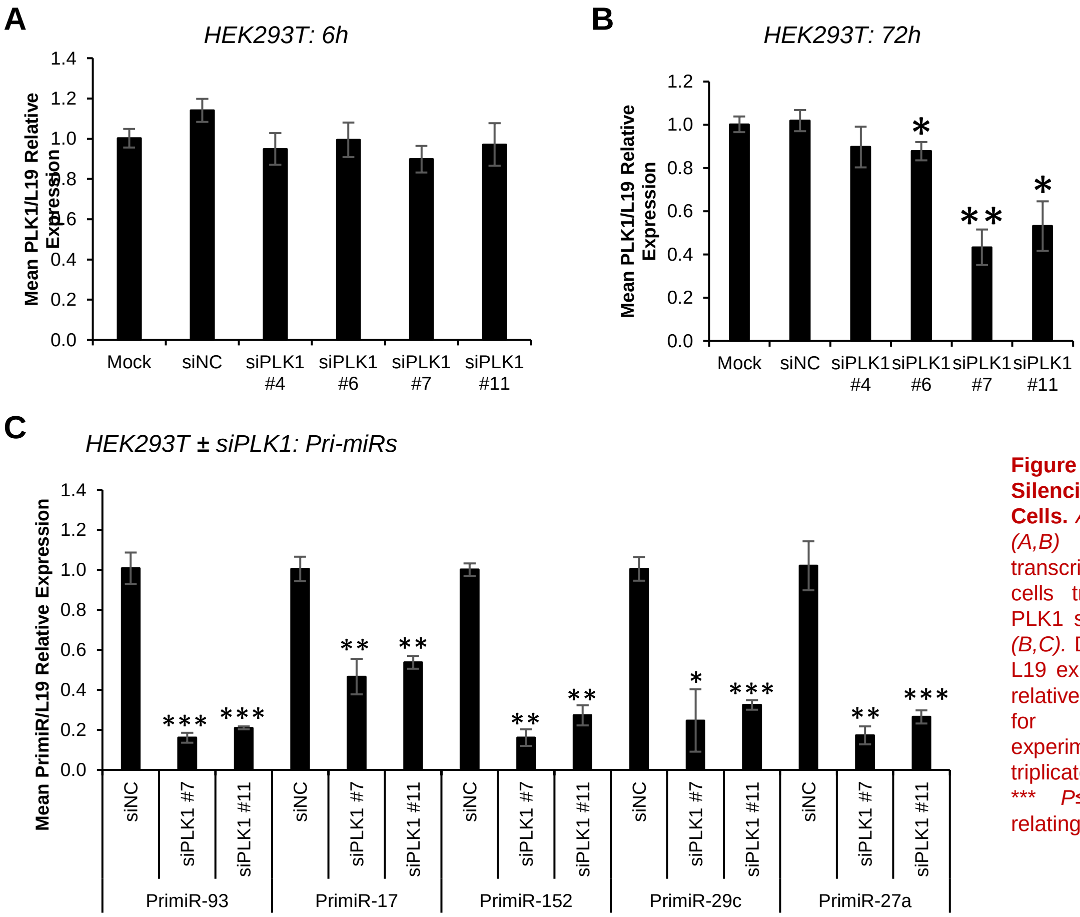

Figure S3: siRNA-Mediated Silencing of PLK1 in HEK293T Cells. *A-C)* qRT-PCR analysis of *(A,B)* PLK1 and *(C)* pri-miR transcript levels in HEK293T cells transfected with different PLK1 siRNAs for 6h *(A)* or 72h *(B,C)*. Data are shown relative to L19 expression. *Columns:* mean relative PLK1 expression ±SEM for three independent experiments performed in triplicate. * *P*≤0.05, ** *P*≤0.005, *** *P*≤0.0001 *vs* NC. Data relating to **Fig 1** are shown.

A

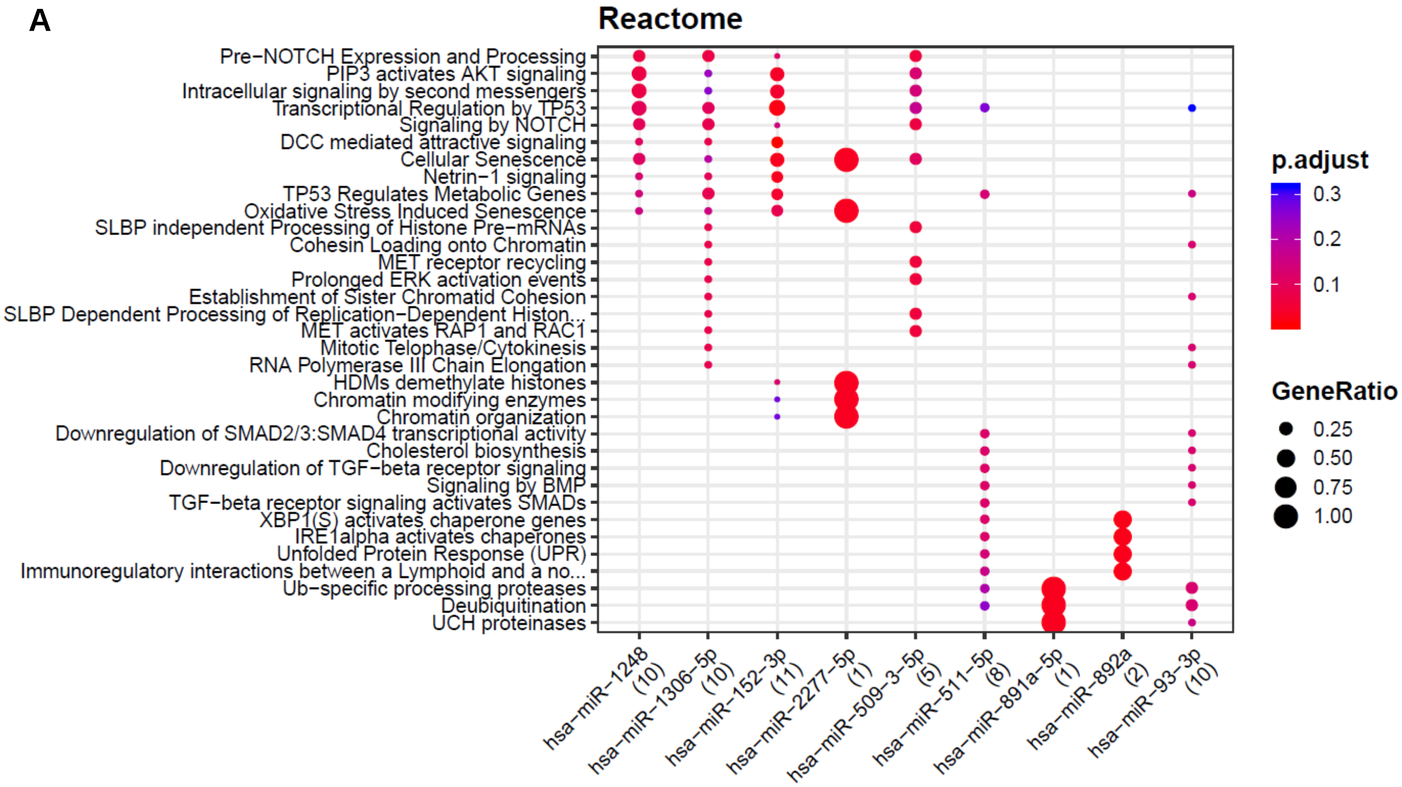

B

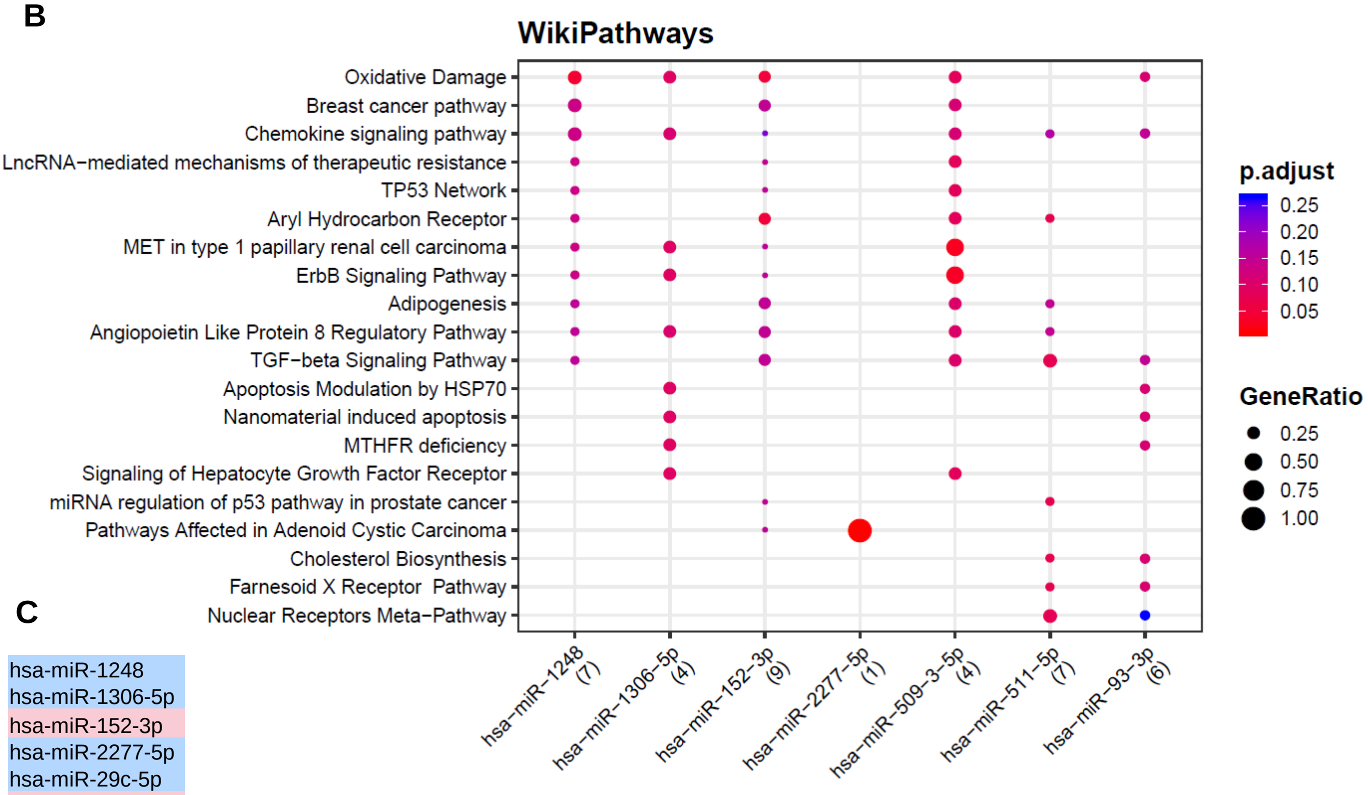

C

hsa-miR-1248
hsa-miR-1306-5p
hsa-miR-152-3p
hsa-miR-2277-5p
hsa-miR-29c-5p
hsa-miR-509-3-5p
hsa-miR-511-5p
hsa-miR-891a-5p
hsa-miR-892a
hsa-miR-93-3p

Figure S4: Pathway Analysis - Shared RO-520- and GSK461-Regulated MicroRNAs. Pathway analysis was conducted using the Mienturnet tool (http://userver.bio.uniroma1.it/apps/mienturnet/) in combination with Reactome *(A)* and WiliPathways *(B)* pathway sets. Only microRNAs for which target information was available were included in the analysis. Shared GSK461- and RO-520-regulated miRs are shown *(C)*. Data relating to **Fig 1** are shown.

Adrenocortical carcinoma

id	Correlation
hsa-mir-4746	0.7432
hsa-mir-130b	0.707
hsa-mir-6783	0.6017
hsa-mir-106b	0.5964
hsa-mir-301b	0.5858
hsa-mir-5003	0.5623
hsa-mir-1246	0.547
hsa-mir-3677	0.544
hsa-mir-3127	0.53
hsa-mir-3170	0.5156

Bladder urothelial carcinoma

id	Correlation
hsa-mir-18a	0.6069
hsa-mir-7-3	0.5583
hsa-mir-942	0.551
hsa-mir-629	0.5413
hsa-mir-1285-1	0.5376
hsa-mir-17	0.5317
hsa-mir-3934	0.5311
hsa-mir-19a	0.5224
hsa-mir-92a-2	0.5127
hsa-mir-92a-1	0.5112

Breast invasive carrcinoma

id	Correlation
hsa-mir-130b	0.6113
hsa-mir-301b	0.6108
hsa-mir-1301	0.5946
hsa-mir-93	0.5746
hsa-mir-301a	0.574
hsa-mir-18a	0.5615
hsa-mir-17	0.5537
hsa-mir-1307	0.5517
hsa-mir-210	0.5454
hsa-mir-106b	0.5444

Cervical squamous cell carcinoma and endo-cervical adenocarcinoma

id	Correlation
hsa-mir-942	0.4858
hsa-mir-18a	0.4798
hsa-mir-130b	0.4436
hsa-mir-1307	0.4415
hsa-mir-17	0.4411
hsa-mir-92a-2	0.4342
hsa-mir-15b	0.4321
hsa-mir-92a-1	0.432
hsa-mir-93	0.4035
hsa-mir-106b	0.398

Cholangiocarcinoma

id	Correlation
hsa-mir-222	0.7692
hsa-mir-92b	0.7686
hsa-mir-21	0.7583
hsa-mir-221	0.7479
hsa-mir-183	0.7326
hsa-mir-330	0.7298
hsa-mir-454	0.7071
hsa-mir-27a	0.7039
hsa-mir-182	0.702
hsa-mir-96	0.6857

Colon adenocarcinoma

id	Correlation
hsa-mir-548d-1	0.432
hsa-mir-590	0.4131
hsa-mir-577	0.4083
hsa-mir-16-2	0.4008
hsa-mir-16-1	0.4006
hsa-mir-1285-1	0.3974
hsa-mir-7-2	0.3889
hsa-mir-301a	0.3871
hsa-mir-7-1	0.3859
hsa-mir-106a	0.3845

Lymphoid Neoplasm Diffuse Large B-cell Lymphoma

id	Correlation
hsa-mir-20a	0.5552
hsa-mir-3662	0.555
hsa-mir-17	0.5115
hsa-mir-573	0.5011
hsa-mir-7974	0.4891
hsa-mir-412	0.4721
hsa-mir-548d-1	0.4559
hsa-mir-19a	0.4462
hsa-mir-496	0.4316
hsa-mir-18a	0.4213

Esophageal carcinoma

id	Correlation
hsa-mir-4746	0.5958
hsa-mir-106b	0.573
hsa-mir-15b	0.5703
hsa-mir-1285-1	0.5653
hsa-mir-18a	0.5544
hsa-mir-942	0.5395
hsa-mir-1268a	0.5384
hsa-mir-196b	0.5369
hsa-mir-1268b	0.5323
hsa-mir-17	0.5308

Glioblastoma multiforme

id	Correlation
hsa-mir-4728	0.9872
hsa-mir-450b	0.9744
hsa-mir-3913-1	0.9559
hsa-mir-3913-2	0.9559
hsa-mir-203a	0.9554
hsa-mir-6767	0.9364
hsa-mir-3176	0.9318
hsa-mir-1269a	0.9314
hsa-mir-7854	0.9239
hsa-mir-4528	0.9201

Head and neck squamous cell carcinoma

id	Correlation
hsa-mir-4746	0.5036
hsa-mir-7-1	0.4736
hsa-mir-1910	0.4733
hsa-mir-21	0.4719
hsa-mir-7-3	0.4592
hsa-mir-1307	0.4548
hsa-mir-196b	0.4547
hsa-mir-18a	0.4497
hsa-mir-15b	0.4494
hsa-mir-130b	0.4422

Kidney chromophobe

id	Correlation
hsa-mir-15b	0.7155
hsa-mir-106b	0.6301
hsa-mir-93	0.6114
hsa-mir-25	0.5588
hsa-mir-16-2	0.5429
hsa-mir-16-1	0.5388
hsa-mir-1285-2	0.5353
hsa-mir-425	0.5335
hsa-mir-130b	0.5246
hsa-mir-3909	0.5169

Kidney renal clear cell carcinoma

id	Correlation
hsa-mir-21	0.68
hsa-mir-155	0.6365
hsa-mir-130b	0.6182
hsa-mir-106b	0.5997
hsa-mir-625	0.5922
hsa-mir-142	0.5771
hsa-mir-4677	0.5349
hsa-mir-25	0.5122
hsa-mir-28	0.5041
hsa-mir-3613	0.4998

Kidney renal papillary cell carcinoma

id	Correlation
hsa-mir-130b	0.5015
hsa-mir-21	0.4896
hsa-mir-4746	0.4879
hsa-mir-106b	0.4737
hsa-mir-17	0.4141
hsa-mir-93	0.4069
hsa-mir-210	0.3888
hsa-mir-4758	0.3831
hsa-mir-4677	0.3811
hsa-mir-584	0.3728

Acute myeloid leukemia

id	Correlation
hsa-mir-324	0.5225
hsa-mir-345	0.5201
hsa-mir-10b	0.4907
hsa-mir-589	0.4434
hsa-mir-185	0.4238
hsa-mir-130b	0.4224
hsa-mir-148a	0.421
hsa-mir-18a	0.4053
hsa-let-7i	0.3969
hsa-mir-214	0.3943

Brain lower grade glioma

id	Correlation
hsa-mir-4746	0.5449
hsa-mir-130b	0.4712
hsa-mir-93	0.4365
hsa-mir-301b	0.4206
hsa-mir-7974	0.4021
hsa-mir-6783	0.3997
hsa-mir-196a-2	0.393
hsa-mir-196a-1	0.3896
hsa-mir-15b	0.3825
hsa-mir-615	0.3665

Liver hepatocellular carcinoma

id	Correlation
hsa-mir-4746	0.73
hsa-mir-106b	0.5842
hsa-mir-3677	0.5749
hsa-mir-93	0.5723
hsa-mir-6783	0.5515
hsa-mir-18a	0.5133
hsa-mir-877	0.5133
hsa-mir-21	0.5127
hsa-mir-1180	0.5084
hsa-mir-589	0.5037

Lung adenocarcinoma

id	Correlation
hsa-mir-1246	0.6075
hsa-mir-130b	0.543
hsa-mir-9-3	0.5262
hsa-mir-9-2	0.5261
hsa-mir-9-1	0.5261
hsa-mir-128-2	0.5179
hsa-mir-128-1	0.5165
hsa-mir-1285-1	0.5103
hsa-mir-548d-1	0.5014
hsa-mir-629	0.4909

Lung squamous cell carcinoma

id	Correlation
hsa-mir-210	0.7014
hsa-mir-130b	0.655
hsa-mir-205	0.6166
hsa-mir-9-1	0.612
hsa-mir-9-2	0.612
hsa-mir-9-3	0.612
hsa-mir-183	0.61
hsa-mir-301b	0.5999
hsa-mir-4652	0.5928
hsa-mir-1268b	0.5902

Mesothelioma

id	Correlation
hsa-mir-6783	0.5818
hsa-mir-130b	0.58
hsa-mir-3662	0.5004
hsa-mir-503	0.4791
hsa-mir-4746	0.459
hsa-mir-1305	0.4249
hsa-mir-21	0.415
hsa-mir-5695	0.4041
hsa-mir-301b	0.4034
hsa-mir-1293	0.3984

Ovarian serous cystadenocarcinoma

id	Correlation
hsa-mir-18a	0.4176
hsa-mir-940	0.4021
hsa-mir-130b	0.3717
hsa-mir-4746	0.3707
hsa-mir-4664	0.3513
hsa-mir-1914	0.3468
hsa-mir-3677	0.3327
hsa-mir-6783	0.3254
hsa-mir-1273a	0.3223
hsa-mir-3200	0.3217

Pancreatic adenocarcinoma

id	Correlation
hsa-mir-135b	0.536
hsa-mir-196b	0.5304
hsa-mir-21	0.5243
hsa-mir-224	0.5139
hsa-mir-584	0.4984
hsa-mir-222	0.4976
hsa-mir-196a-1	0.4902
hsa-mir-210	0.4893
hsa-mir-196a-2	0.4868
hsa-mir-106b	0.4761

Pheochromocytoma and Paraganglioma

id	Correlation
hsa-mir-210	0.4067
hsa-mir-130b	0.3308
hsa-mir-301a	0.3147
hsa-mir-4691	0.2963
hsa-mir-378f	0.2721
hsa-mir-1226	0.2708
hsa-mir-4746	0.2624
hsa-mir-301b	0.2561
hsa-mir-3179-1	0.248
hsa-mir-3179-3	0.248

Prostate adenocarcinoma

id	Correlation
hsa-mir-15b	0.5625
hsa-mir-425	0.5577
hsa-mir-93	0.5088
hsa-mir-191	0.5029
hsa-mir-21	0.5023
hsa-mir-25	0.4976
hsa-mir-183	0.4925
hsa-mir-106b	0.4786
hsa-mir-96	0.4737
hsa-mir-182	0.4631

Rectum adenocarcinoma

id	Correlation
hsa-mir-1285-2	0.6078
hsa-mir-1285-1	0.5945
hsa-mir-577	0.585
hsa-mir-203b	0.5575
hsa-mir-19a	0.5484
hsa-mir-3677	0.5432
hsa-mir-203a	0.5424
hsa-mir-106a	0.5316
hsa-mir-130b	0.5197
hsa-mir-628	0.5112

Sarcoma

id	Correlation
hsa-mir-4746	0.6776
hsa-mir-130b	0.5948
hsa-mir-93	0.5914
hsa-mir-15b	0.5744
hsa-mir-106b	0.5426
hsa-mir-942	0.5097
hsa-mir-18a	0.494
hsa-mir-210	0.4925
hsa-mir-6783	0.4735
hsa-mir-454	0.4708

Skin cutaneous melanoma

id	Correlation
hsa-mir-4746	0.5606
hsa-mir-130b	0.5068
hsa-mir-1246	0.4592
hsa-mir-3682	0.4531
hsa-mir-942	0.4437
hsa-mir-7974	0.4399
hsa-mir-6783	0.4229
hsa-mir-15b	0.4041
hsa-mir-3662	0.3855
hsa-mir-4440	0.3771

Stomach adenocarcinoma

id	Correlation
hsa-mir-130b	0.7276
hsa-mir-106b	0.6995
hsa-mir-942	0.6892
hsa-mir-183	0.6753
hsa-mir-18a	0.6705
hsa-mir-200a	0.6655
hsa-mir-429	0.6615
hsa-mir-222	0.651
hsa-mir-1307	0.6484
hsa-mir-200b	0.6474

Testicular germ cell tumours

id	Correlation
hsa-mir-302c	0.7252
hsa-mir-3937	0.7245
hsa-mir-367	0.7079
hsa-mir-522	0.7001
hsa-mir-302a	0.6926
hsa-mir-1283-1	0.6731
hsa-mir-1283-2	0.6731
hsa-mir-516a-2	0.6666
hsa-mir-516a-1	0.6666
hsa-mir-519a-1	0.6643

Thyroid carcinoma

id	Correlation
hsa-mir-155	0.5736
hsa-mir-4491	0.5627
hsa-mir-142	0.5138
hsa-mir-625	0.4681
hsa-mir-150	0.4535
hsa-mir-3150b	0.4501
hsa-mir-5571	0.4338
hsa-mir-766	0.4323
hsa-mir-342	0.4302
hsa-mir-7702	0.4167

Thymoma

id	Correlation
hsa-mir-942	0.913
hsa-mir-106a	0.9052
hsa-mir-4746	0.8969
hsa-mir-548d-1	0.8835
hsa-mir-196b	0.8833
hsa-mir-18a	0.8798
hsa-mir-130b	0.8747
hsa-mir-18b	0.8746
hsa-mir-548d-2	0.8722
hsa-mir-93	0.8697

Uterine corpus endometrial carcinoma

id	Correlation
hsa-mir-130b	0.6376
hsa-mir-15b	0.6355
hsa-mir-1307	0.6152
hsa-mir-18a	0.6096
hsa-mir-301b	0.5741
hsa-mir-183	0.5656
hsa-mir-877	0.564
hsa-mir-548d-1	0.5632
hsa-mir-942	0.5632
hsa-mir-1246	0.5618

Uterine carcinosarcoma

id	Correlation
hsa-mir-93	0.4409
hsa-mir-25	0.4338
hsa-mir-5585	0.4273
hsa-mir-1272	0.4159
hsa-mir-106b	0.4132
hsa-mir-561	0.4026
hsa-mir-33a	0.3855
hsa-mir-7974	0.379
hsa-mir-15b	0.369
hsa-mir-523	0.3627

Uveal melanoma

id	Correlation
hsa-mir-6783	0.4351
hsa-let-7b	0.4106
hsa-mir-937	0.4086
hsa-mir-548d-1	0.3778
hsa-mir-362	0.3694
hsa-mir-670	0.3593
hsa-mir-1254-1	0.3566
hsa-mir-1254-2	0.3558
hsa-mir-675	0.3494
hsa-let-7a-3	0.3477

Figure S5: Top 10 MiRs Correlating with PLK1 Expression in Cancers of the TCGA Data Set. Small RNA-seq-/ qRT-PCR-identified PLK1-regulated miRs are highlighted in blue boxes. Correlation refers to Pearson's correlation coefficient. Data relating to **Fig 1** are shown.

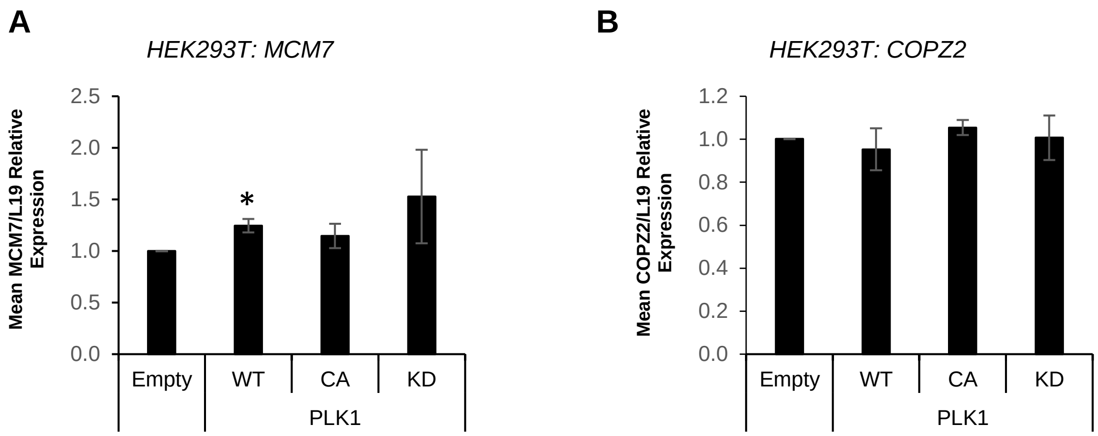

Figure S6: PLK1 does not Modulate Host Genes of Intronic PLK1-Regulated MiRs. PLK1-regulated miR-93-3p is found within an intron of protein-coding MCM7 gene, whilst PLK1-regulated miR-152 is within an intron of COPZ2. *A,B)* qRT-PCR analysis of *A)* MCM7 and *B)* COPZ2 transcript levels in HEK293T cells transfected with empty plasmid, PLK1 WT, PLK1-T^{210}D (constitutively-active) or PLK1-K^{82}R (kinase-dead) for 72h. *Columns:* mean relative transcript levels normalised to L19 for three independent experiments performed in triplicate ± SEM. * $P \leq 0.05$ *vs* empty plasmid control. Data relating to **Fig 2** are shown.

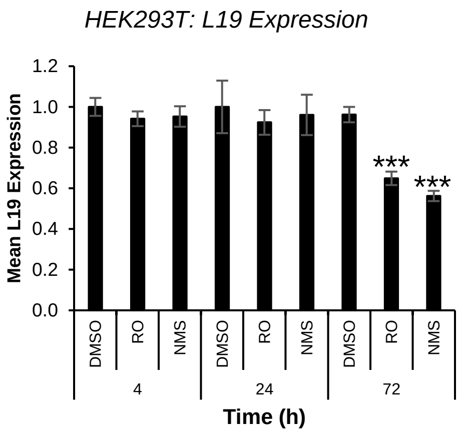

Figure S7: PLK1 Inhibition Decreases L19 Expression at 72h. qRT-PCR analysis of L19 transcript levels in HEK293T cells treated with DMSO, RO-5203280 (100nM) or NMS1286 (500nM) for 4, 24 or 72h. *Columns:* mean relative L19 transcript levels for three independent experiments performed in triplicate ± SEM. *** *P* ≤ 0.0001 *vs* DMSO control. Data relating to **Fig 2** are shown.

A

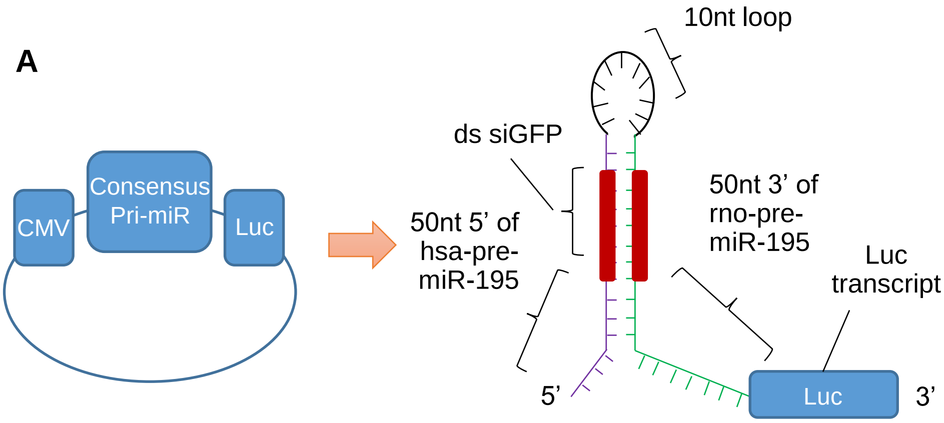

B

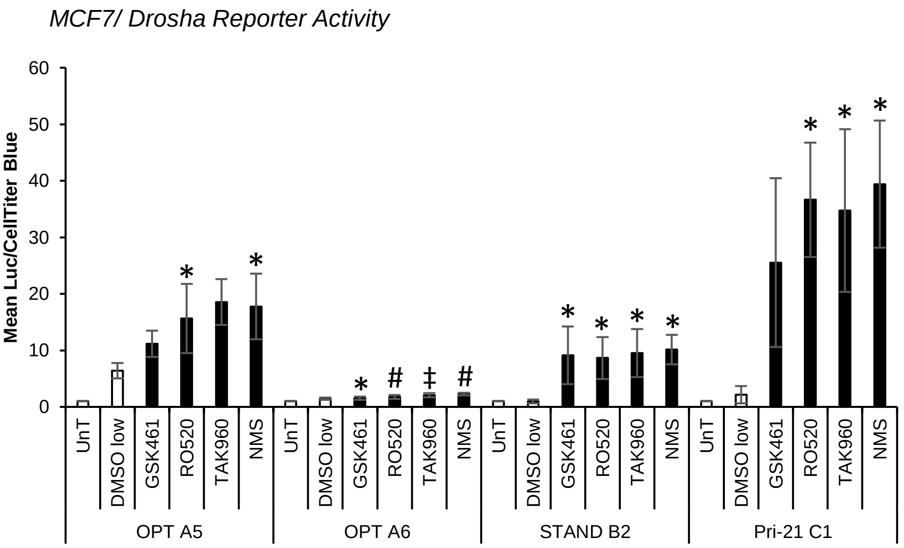

Figure S8: PLK1 Inhibitors Reduce Activity of Luciferase-Based Drosha Reporters. *A)* Schematic illustration of Drosha activity reporter constructs used for luciferase assays. CMV promoter drives transcription of the consensus pri-miR linked to luciferase. Drosha cleavage of the pri-miR results in loss of luciferase transcript and thus reduced luciferase activity. Hence luminescence is inversely proportional to Drosha activity. *B)* Luciferase assay analysis of MCF7 monoclonal cell lines stably expressing above Drosha reporter constructs and treated with PLK1 inhibitors (GSK461, RO-520, TAK960 – 100nM, NMS – 500nM) for 96h. Luciferase activity is inversely proportional to Drosha activity and was corrected for PLK1 inhibitor effects on cell number by CellTiter Blue assay. *Columns:* mean ± SEM for three independent experiments performed in quadruplicate. OPT = optimal universal reporter, STAND = standard reporter, pri-21 = pri-miR-21-specific reporter (see also **Materials and Methods**). * $P \leq 0.05$, # $P \leq 0.005$, ‡ $P \leq 0.0001$. Data relating to **Fig 2** are shown.

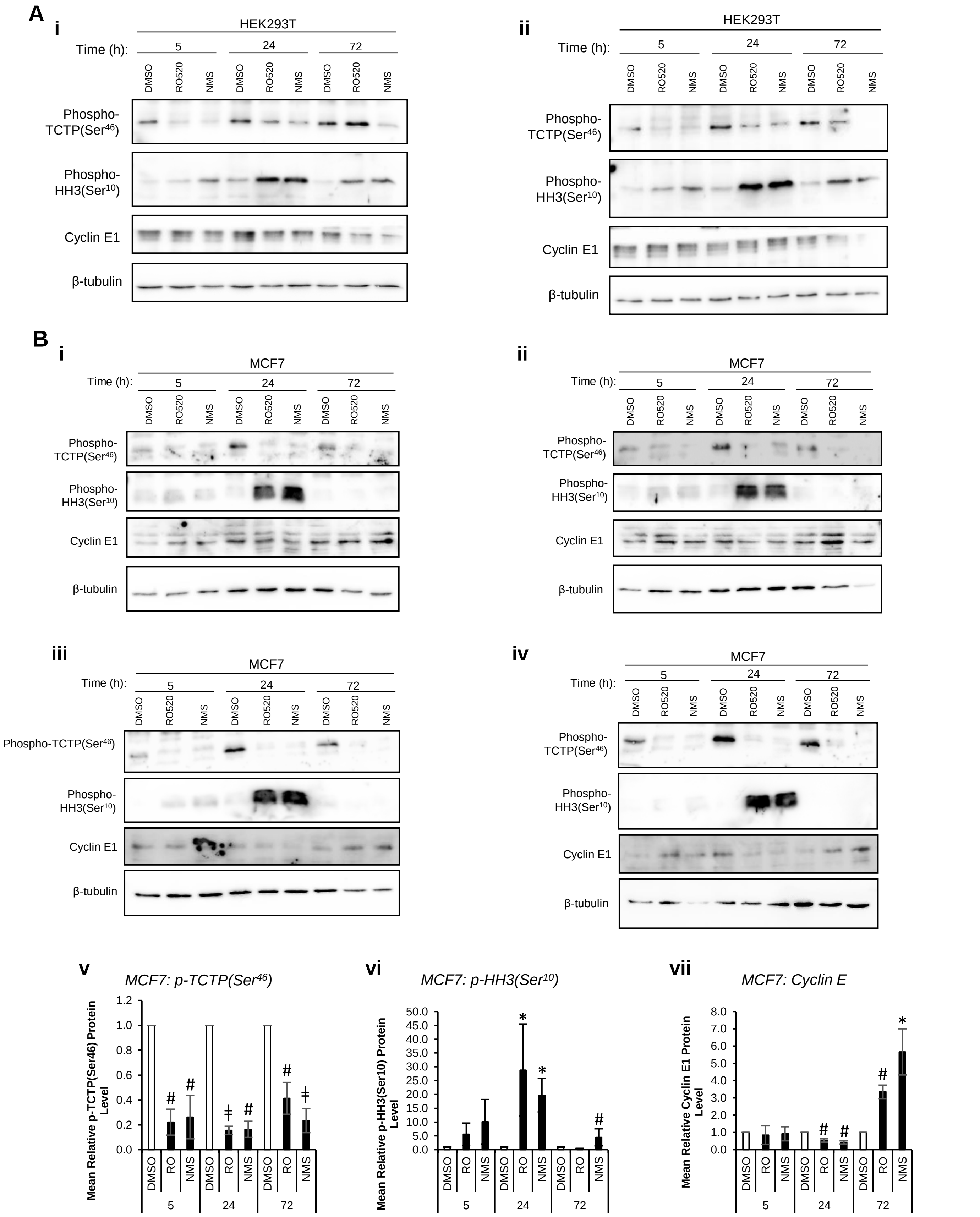

Figure S9: PLK1 Inhibition Leads to M-Phase Cell Cycle Arrest at 24h and 72h, but not 5h, in HEK293T and MCF7 Cells. Western blot analysis of phospho-TCTP(Ser46), phospho-Histone H3 (Ser10) and cyclin E1 protein levels in *(A)* HEK293T and *(B)* MCF7 cells treated with DMSO, RO-520 (100nM) or NMS (500nM) for 5, 24 or 72h. β-tubulin was used as a control for loading. *(A)* Independent biological repeats relating to **Fig 2** are shown. *Bv,vi,vii)* Densitometry was performed using ImageJ. *Columns:* mean relative protein level for three independent biological repeats relative to DMSO control ± SEM. Data relating to **Fig 2** are shown. * $P \leq 0.05$, # $P \leq 0.005$, ǂ $P \leq 0.0001$.

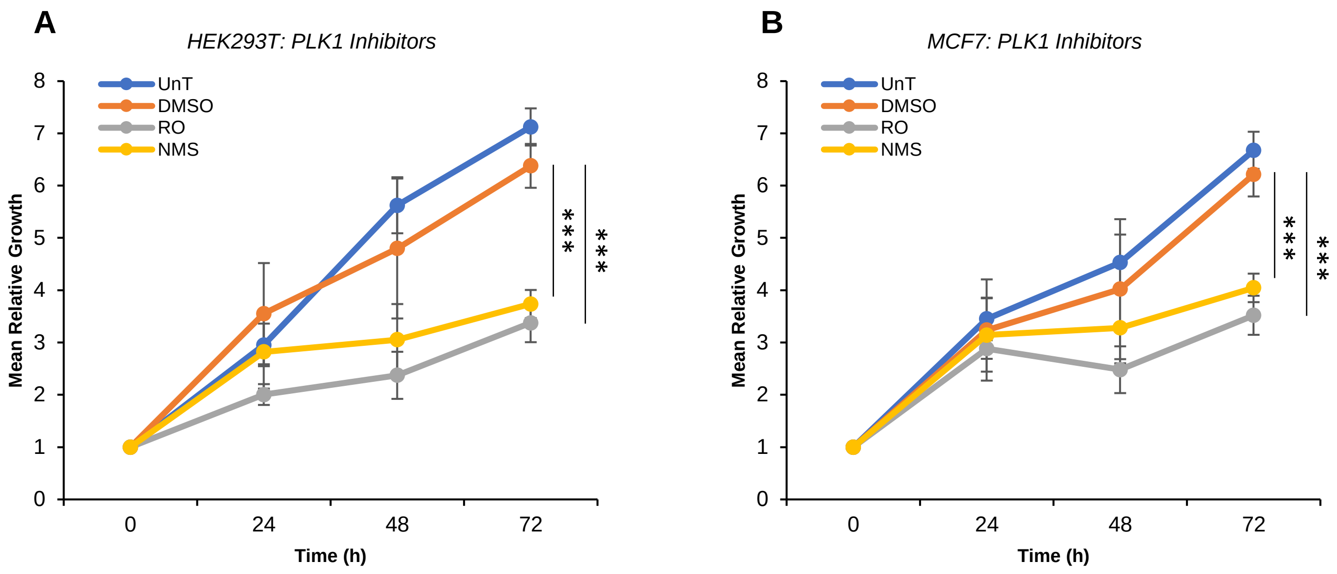

Figure S10: Treatment of HEK293T and MCF7 Cells with PLK1 Inhibitors Significantly Reduces Cell Proliferation. *A,B)* Sulphorhodamine B assay analysis of proliferation of *(A)* HEK2393T and *(B)* MCF7 cells treated ± DMSO, RO-5203280 (100nM) or NMS1286 (500nM) for 0-72h. Data are shown relative to cell number at day 0 and represent mean ± SEM for three independent experiments performed in quadruplicate. *** *P*≤0.0001. Data relating to **Fig 2** are shown.

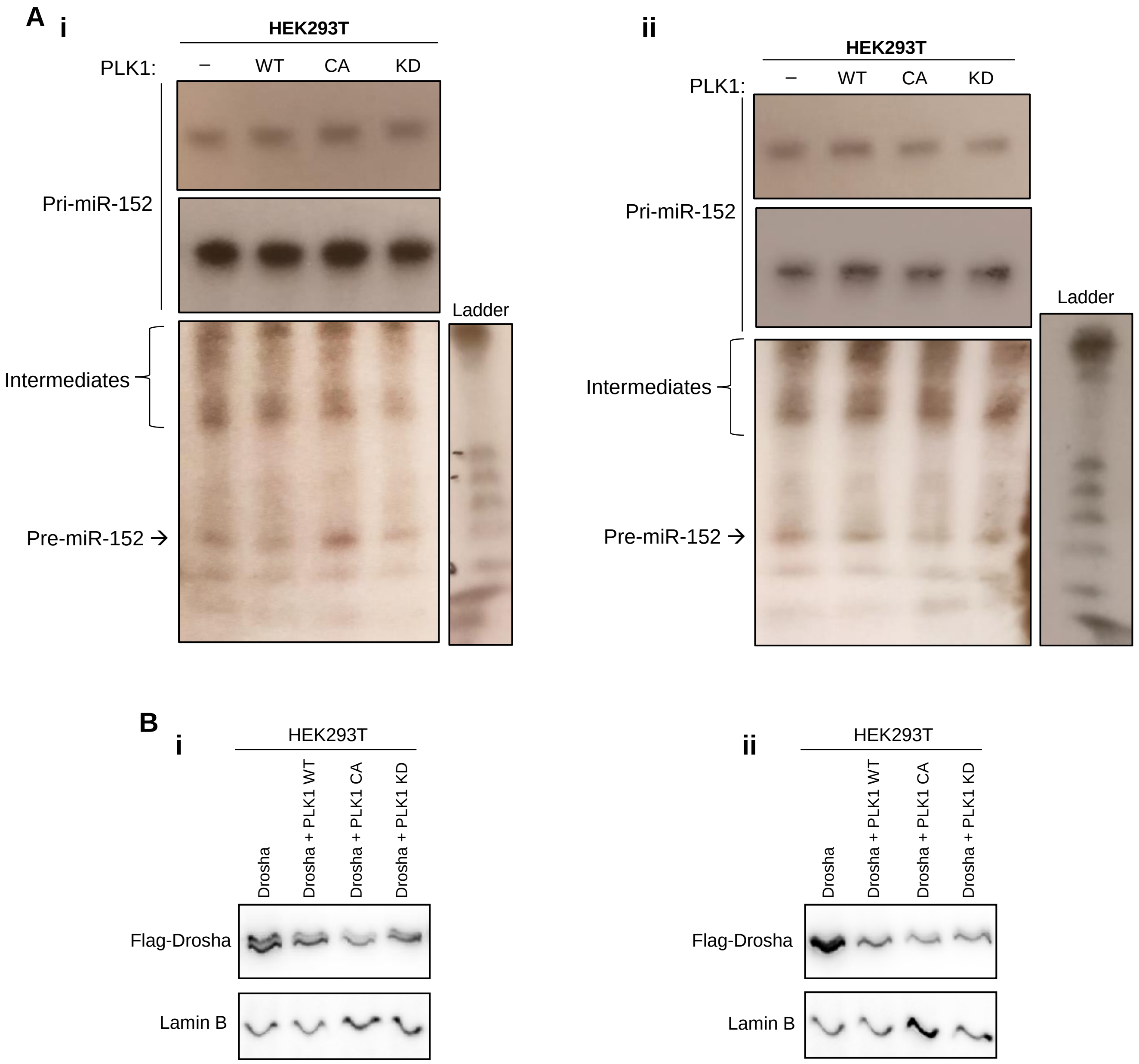

Figure S11: PLK1 Inhibition Reduces Microprocessor-Mediated Pri-miR-152 to Pre-miR-152 Processing. *In vitro* pri-miR processing assay analysis of Drosha activity in response to PLK1 inhibition. *In vitro*-transcribed, ^{32}P radio-labelled pri-miR-152 sequence was incubated with Flag-Drosha immunoprecipitates of HEK293T cells transfected with Flag-Drosha ± WT, constitutively-active ($T^{210}D$) and kinase-dead ($K^{82}R$) PLK1 for 48h. N=2 biological replicates relating to **Fig 3C** are shown. For quantification, data were normalised to Flag-Drosha input corrected for loading (from *(B)*). *B)* Western blot analysis of Drosha protein levels in lysates of HEK293T cells transfected with Flag-Drosha ± WT, constitutively-active ($T^{210}D$) and kinase-dead ($K^{82}R$) PLK1 for 48h from above. Lamin B was used as a control for loading.

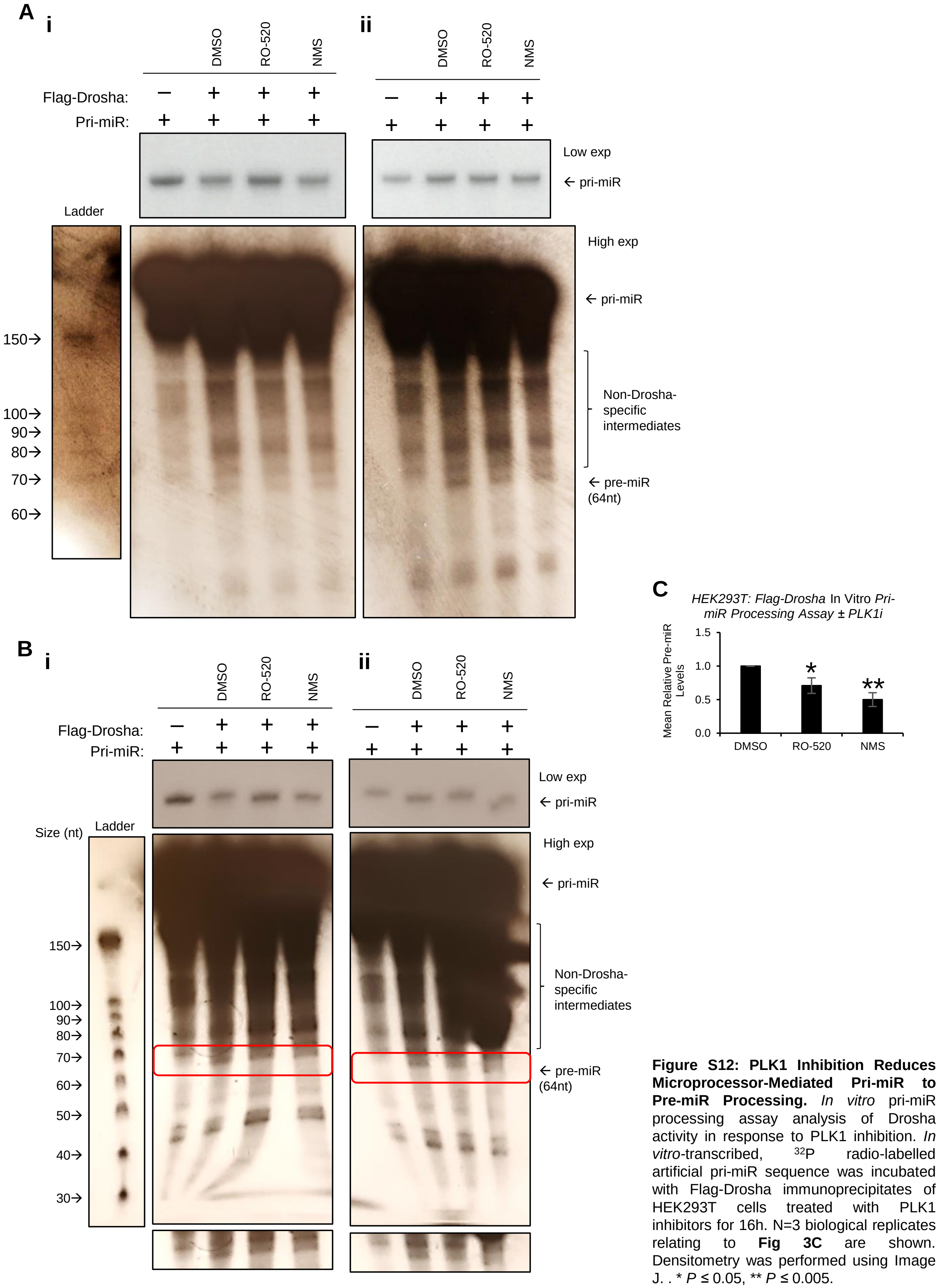

Figure S12: PLK1 Inhibition Reduces Microprocessor-Mediated Pri-miR to Pre-miR Processing. *In vitro* pri-miR processing assay analysis of Drosha activity in response to PLK1 inhibition. *In vitro*-transcribed, ^{32}P radio-labelled artificial pri-miR sequence was incubated with Flag-Drosha immunoprecipitates of HEK293T cells treated with PLK1 inhibitors for 16h. N=3 biological replicates relating to **Fig 3C** are shown. Densitometry was performed using Image J. . * *P* ≤ 0.05, ** *P* ≤ 0.005.

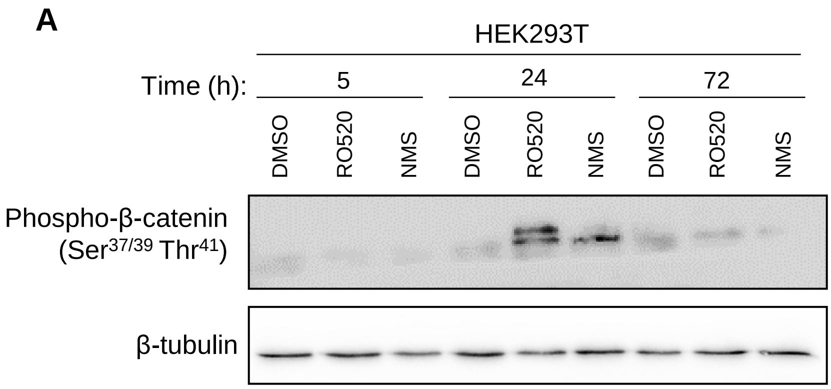

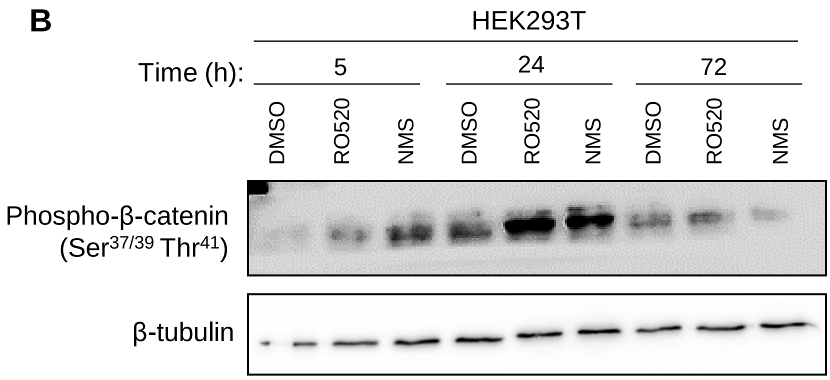

Figure S13: Impact of PLK1 Inhibitor Treatment on GSK3β Activity. *A,B)* Western blot analysis of phospho-β-catenin ($Ser^{37/39}$ Thr^{41}) protein levels in HEK293T cells treated with DMSO, RO-520 (100nM) or NMS (500nM) for 4, 24 and 72h. β-tubulin was used as a loading control. Independent biological repeats relating to **Fig 3** are shown.

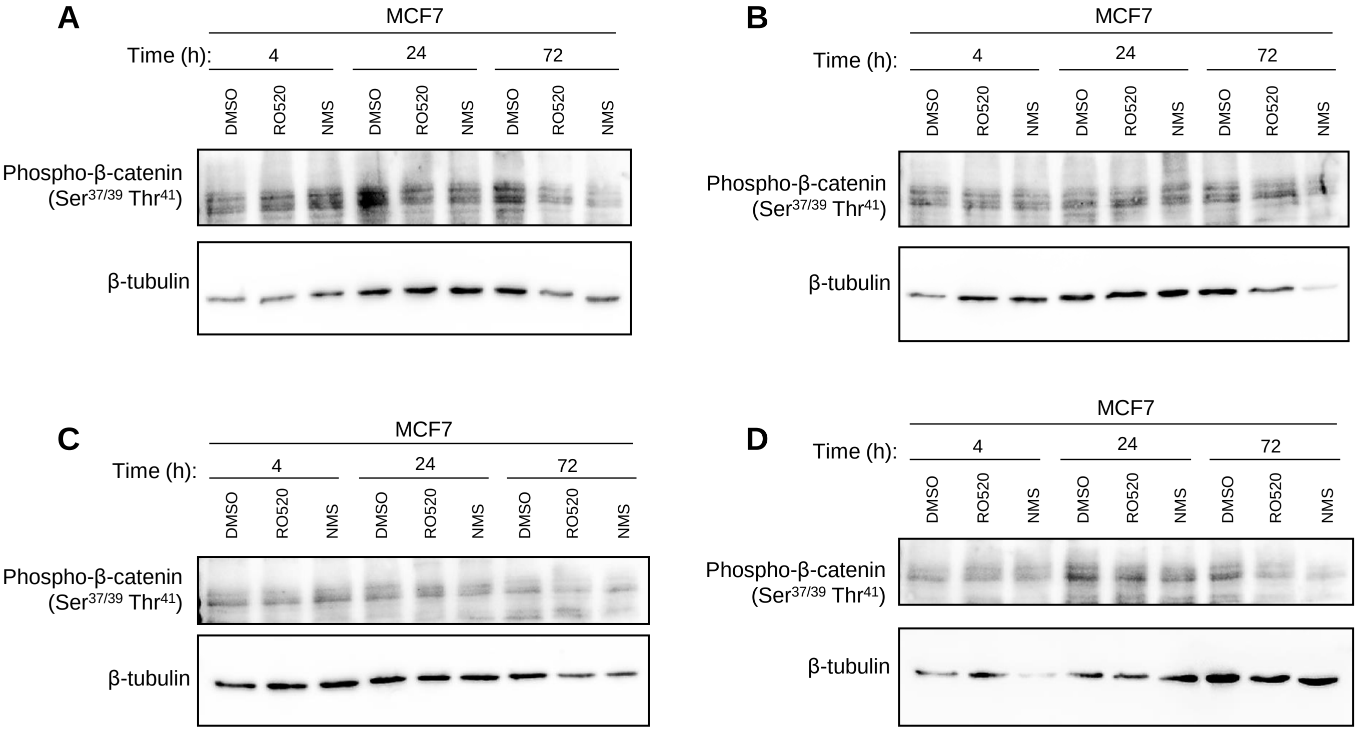

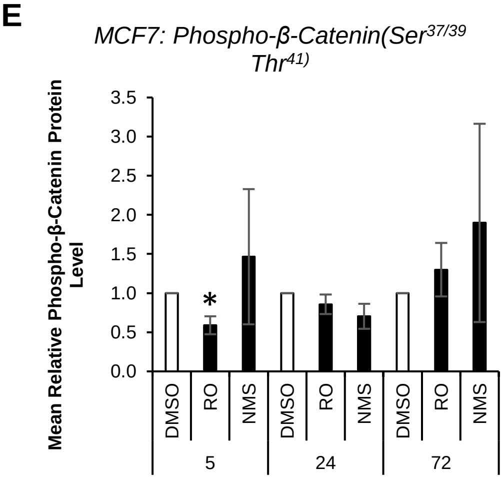

Figure S14: Impact of PLK1 Inhibition on GSK3β Activity in MCF7 Cells. Western blot analysis of phospho-β-catenin ($Ser^{37/39}$ Thr^{41}) protein levels in MCF7 cells treated with DMSO, RO-520 (100nM) or NMS (500nM) for 4, 24 and 72h. β-tubulin was used as a loading control. *E)* Densitometry was performed using ImageJ. *Columns:* mean relative protein levels relative to β-tubulin ±SEM (n=4). See also **Fig 3**.

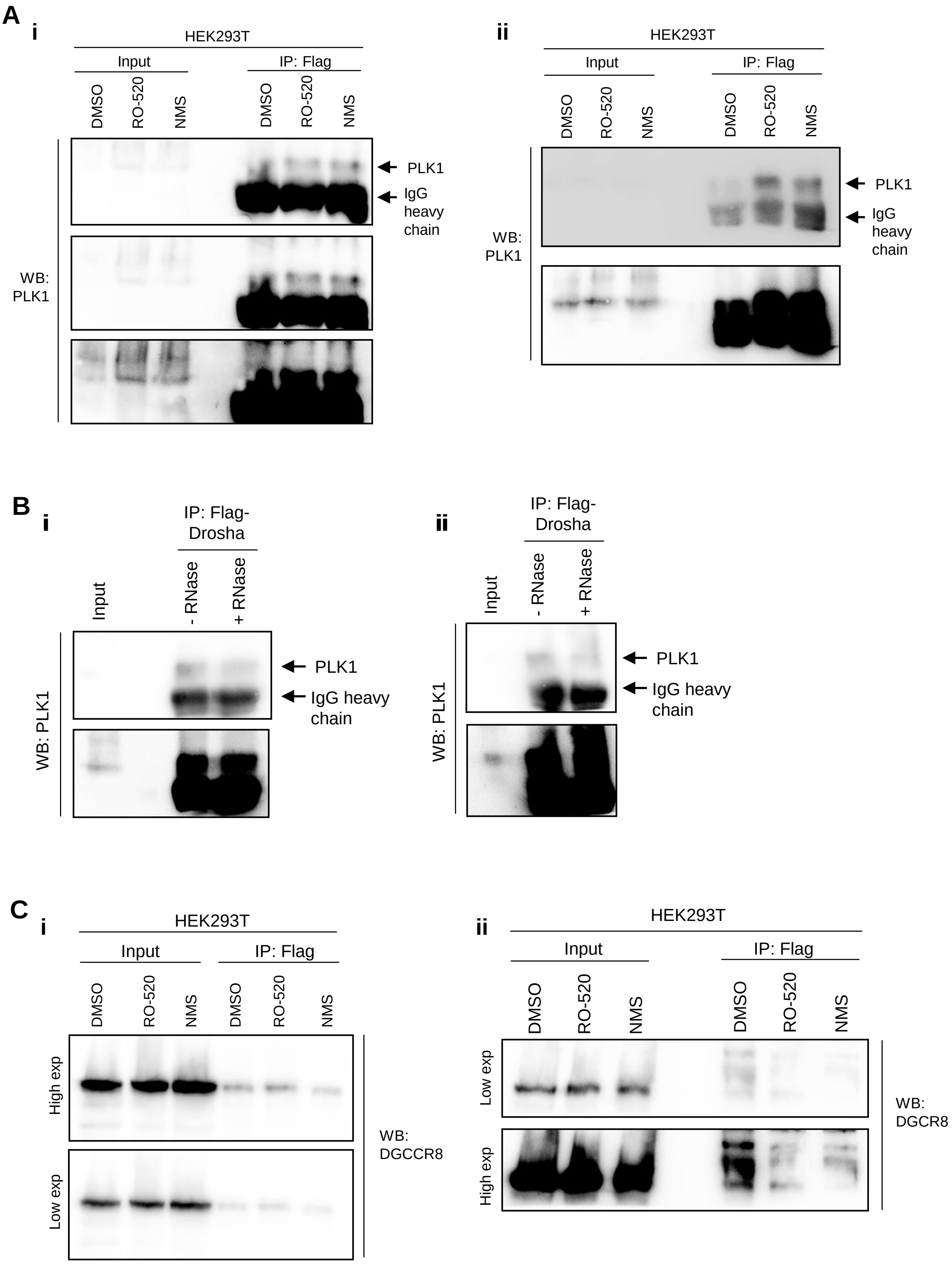

Figure S15: PLK1 Interacts with Drosha in an RNA-Dependent Manner and Modulates its Association with DGCR8. Western blot analysis of *A,B)* PLK1 and *C)* DGCR8 protein levels in Flag immunoprecipitates of HEK293T cells transfected with Flag-Drosha and treated with DMSO, RO-520 (100nM) or NMS (500nM) for 16h *(A,C)*, or with RNase A *(B)*. Independent biological repeats relating to **Fig 4A, B and C** are shown.

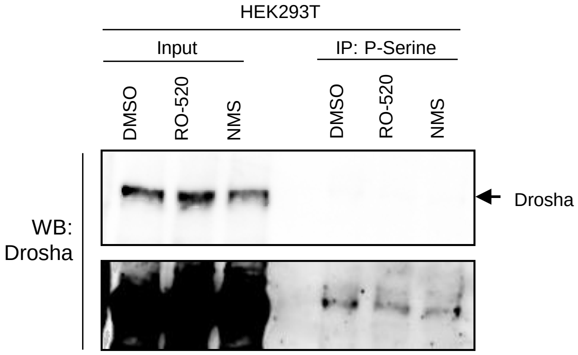

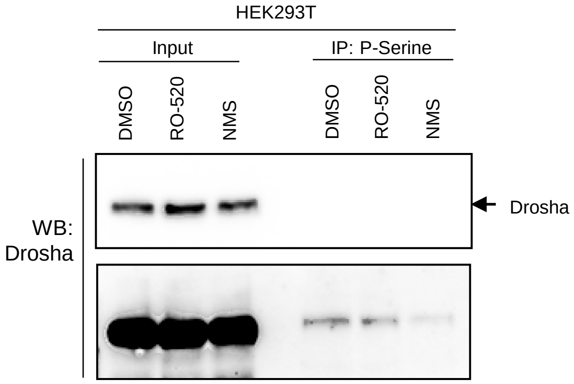

Figure S16: PLK1 Modulates Drosha Phosphorylation. Western blot analysis of Drosha protein levels in phospho-serine immunoprecipitates of HEK293T cells treated with DMSO, RO-520 (100nM) or NMS (500nM) for 16h. Independent biological repeats relating to **Fig 4D** are shown.

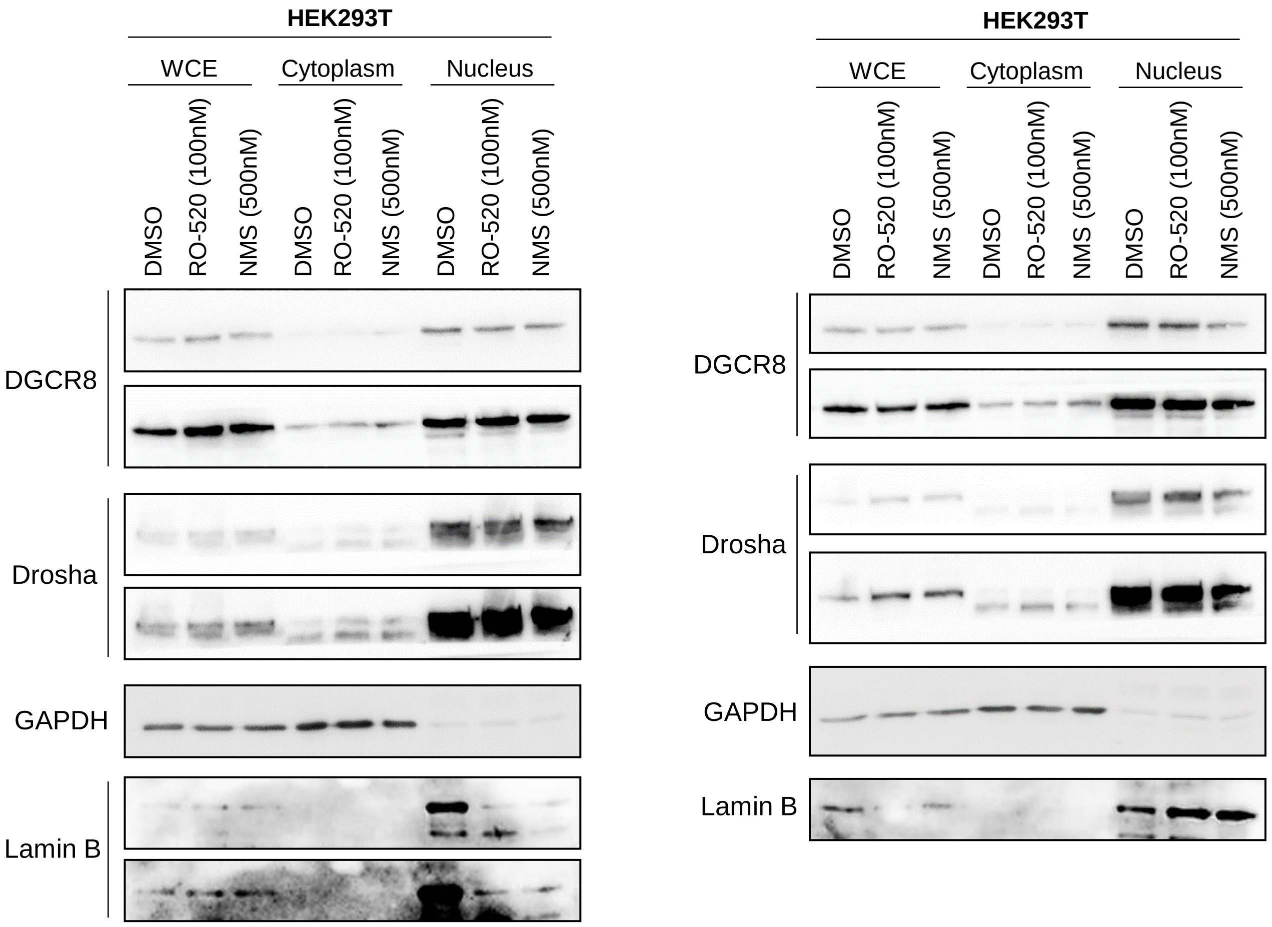

Figure S17: Short-Term PLK1 Inhibition Modulates Drosha and DGCR8 Subcellular Localisation. Western blot analysis of DGCR8 and Drosha protein levels in whole cell extracts (WCEs), cytoplasmic and nuclear fractions of HEK293T cells treated with DMSO, RO-520 (100nM) or NMS (500nM) for 3h. GAPDH and Lamin B were used as cytoplasmic and nuclear controls, respectively. Independent biological repeats relating to **Fig 4E** are shown.

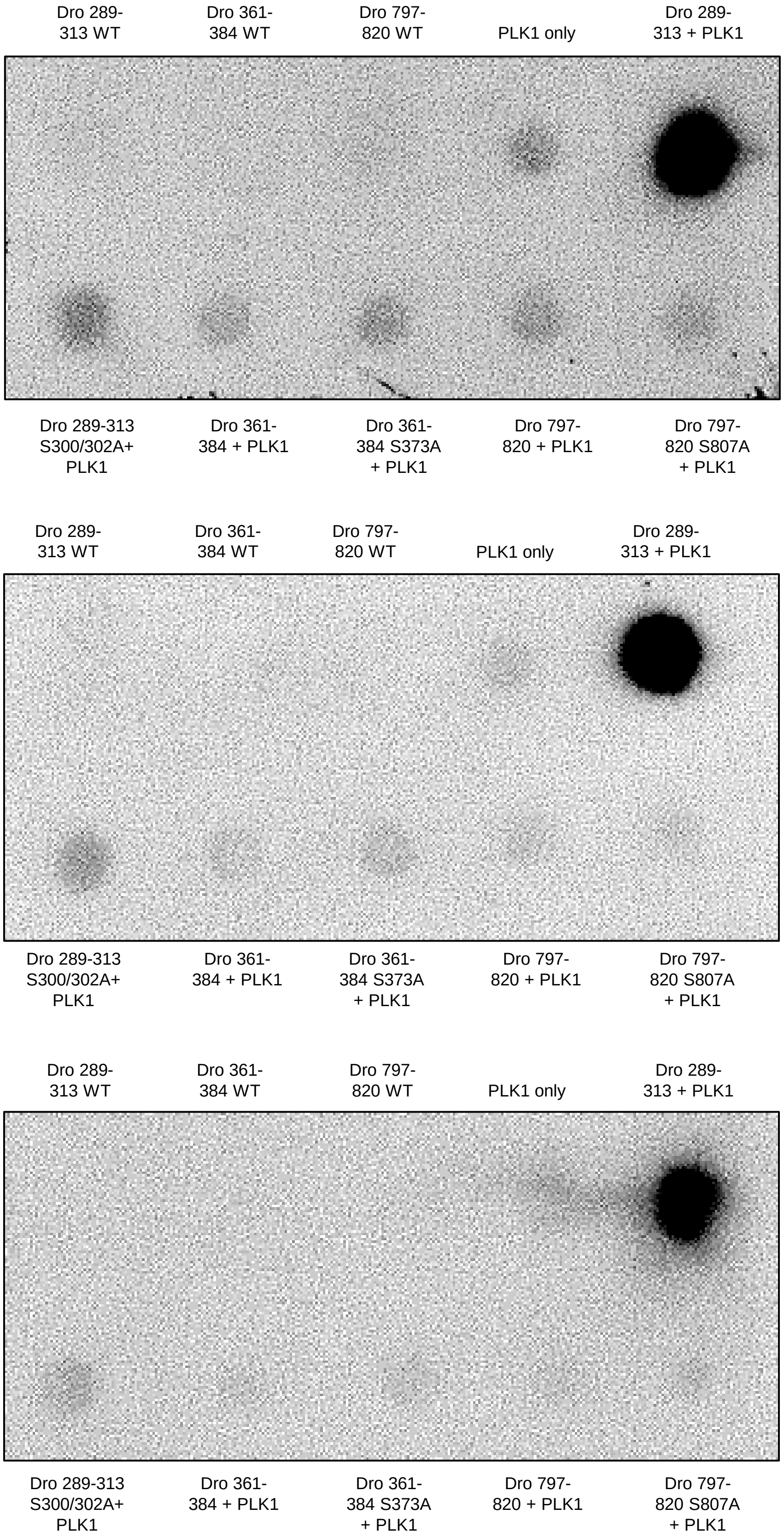

Figure S18: PLK1 Phosphorylates Drosha at S^{300} and/or S^{302}. *In vitro* kinase assay analysis of PLK1 phosphorylation of WT and phospho site-mutant Drosha peptides. Indicated Drosha peptides were incubated with recombinant PLk1 in the presence of α-^{32}P-ATP. Independent biological repeats relating to **Fig 5A** are shown.

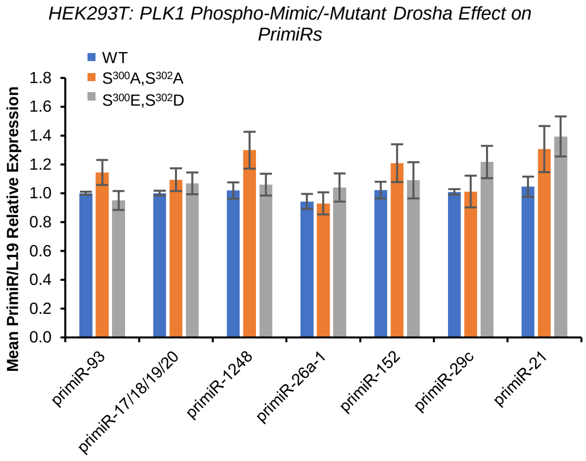

Figure S19: PLK1 Phosphorylation of Drosha at S^{300} and/or S^{302} May Alter Pri-miR Levels. qRT-PCR analysis of pri-miR levels in HEK293T cells transfected with WT, $S^{300}A,^{302}A$ or $S^{300}E,^{302}D$ Flag-Drosha for 72h. L19 was used for normalisation. *Columns:* mean pri-miR levels for three independent experiments performed in triplicate ±SEM. Data relating to **Fig 5** are shown.

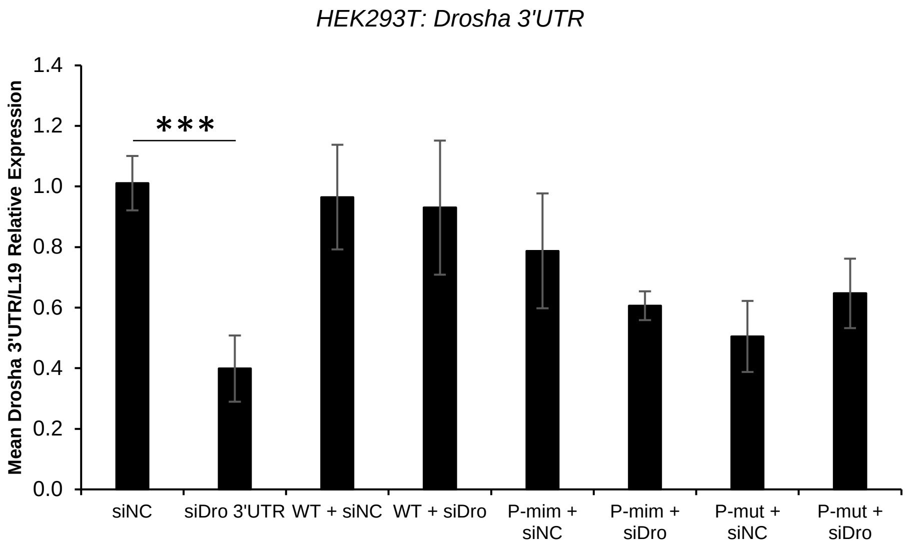

Figure S20: siRNA Targeting Drosha 3'UTR Significantly Reduces Endogenous, but not siRNA-Resistant Exogenous, Drosha Transcript Levels. qRT-PCR analysis of Drosha transcript levels in HEK293T cells transfected with Drosha 3'UTR siRNA ± WT, phospho-mimic (P-mim – S300E,S302D) or phospho-mutant (P-mut – S300A,S302A) Flag-Drosha for 72h. *Columns:* mean relative Drosha expression normalised to L19 ± SEM for three independent experiments performed in triplicate. *** $P \leq 0.0001$.

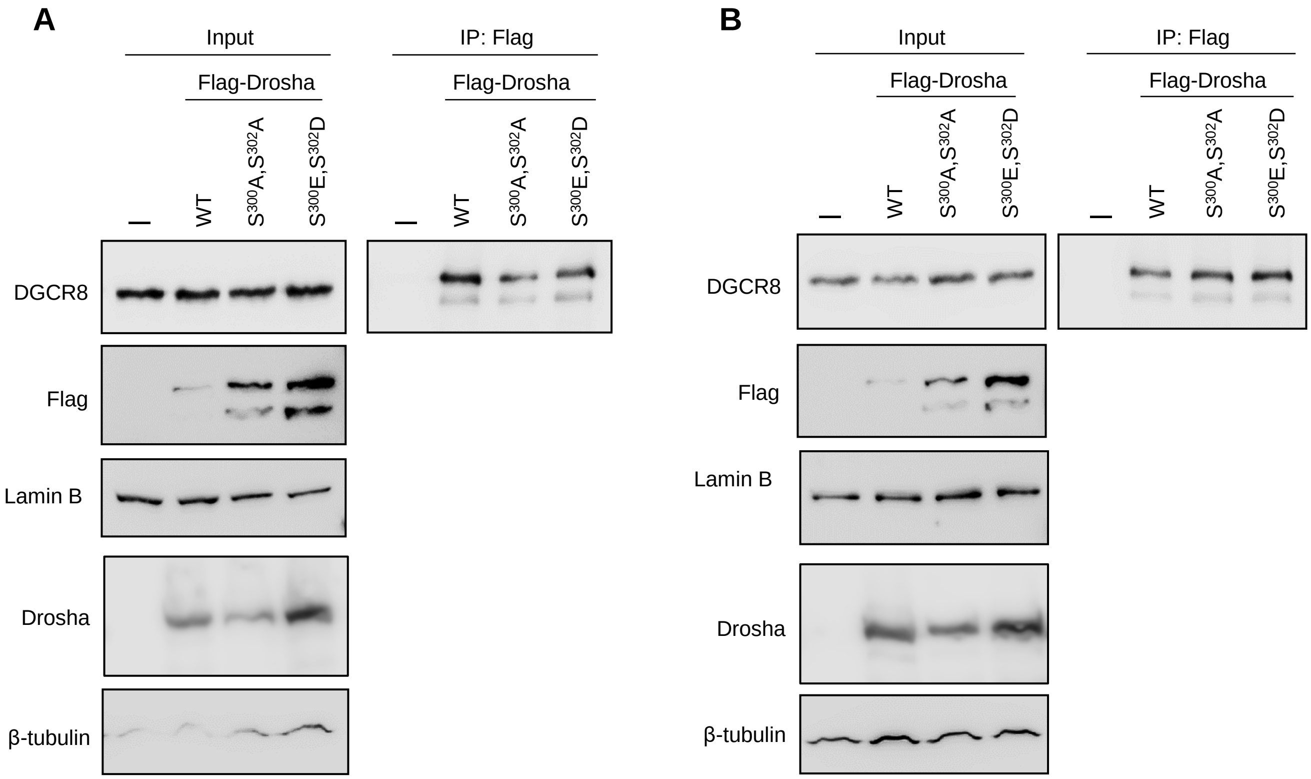

Figure S21: PLK1 Phosphorylation of Drosha at S^{300} and/or S^{302} Alters Drosha:DGCR8 Interaction. Western blot analysis of DGCR8 protein levels in Flag immunoprecipitates of HEK293T cells transfected with WT, phospho-mutant ($S^{300}A,S^{302}A$) or phospho-mimic ($S^{300}E,S^{302}D$) for 72h. Biological repeats relating to **Fig 5D** are shown. Immunoprecipitated DGCR8 was normalised to input levels of loading control-corrected Drosha (bottom panels – longer exposures in **Fig S22**).

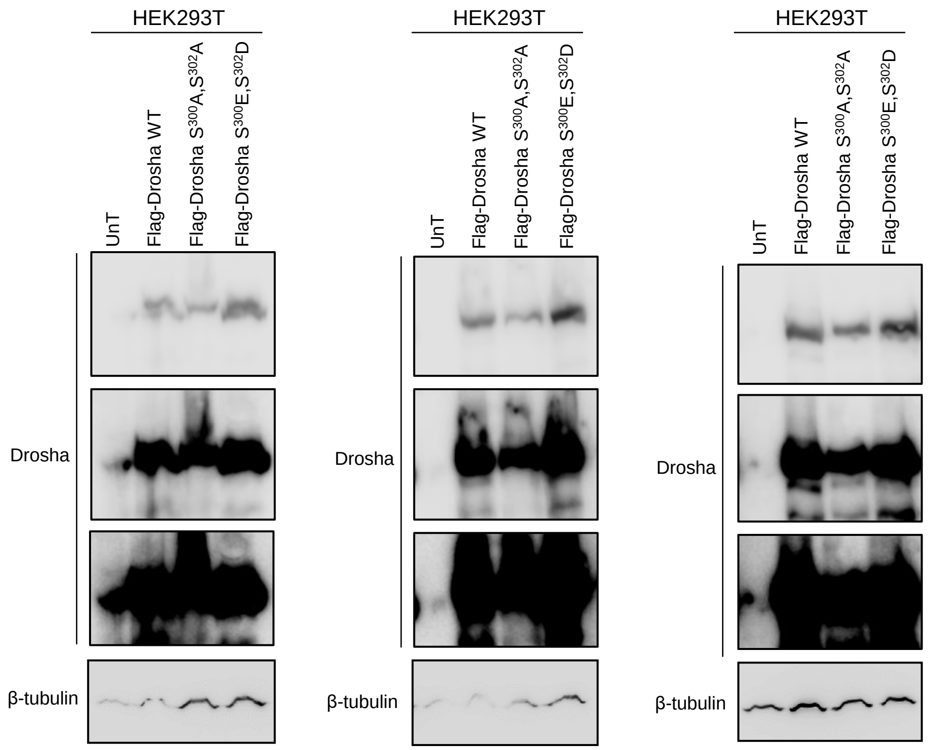

Figure S22: Drosha Protein Levels in Lysates of HEK293T Cells Transfected with WT, Phospho-Mutant or Phospho-Mimic Drosha. Western blot analysis of Drosha protein levels in HEK293T cells transfected with pCK-Flag-Drosha WT, S300A,S302A phospho-mutant or S300E,S302D phospho-mimic for 72h. β-tubulin was used as a control for loading and densitometry was performed using ImageJ. Longer exposures are shown to permit visualisation of endogenous Drosha. *Columns:* mean relative Drosha protein levels normalised to β-tubulin ± SEM for three independent experiments. * $P \leq 0.05$.

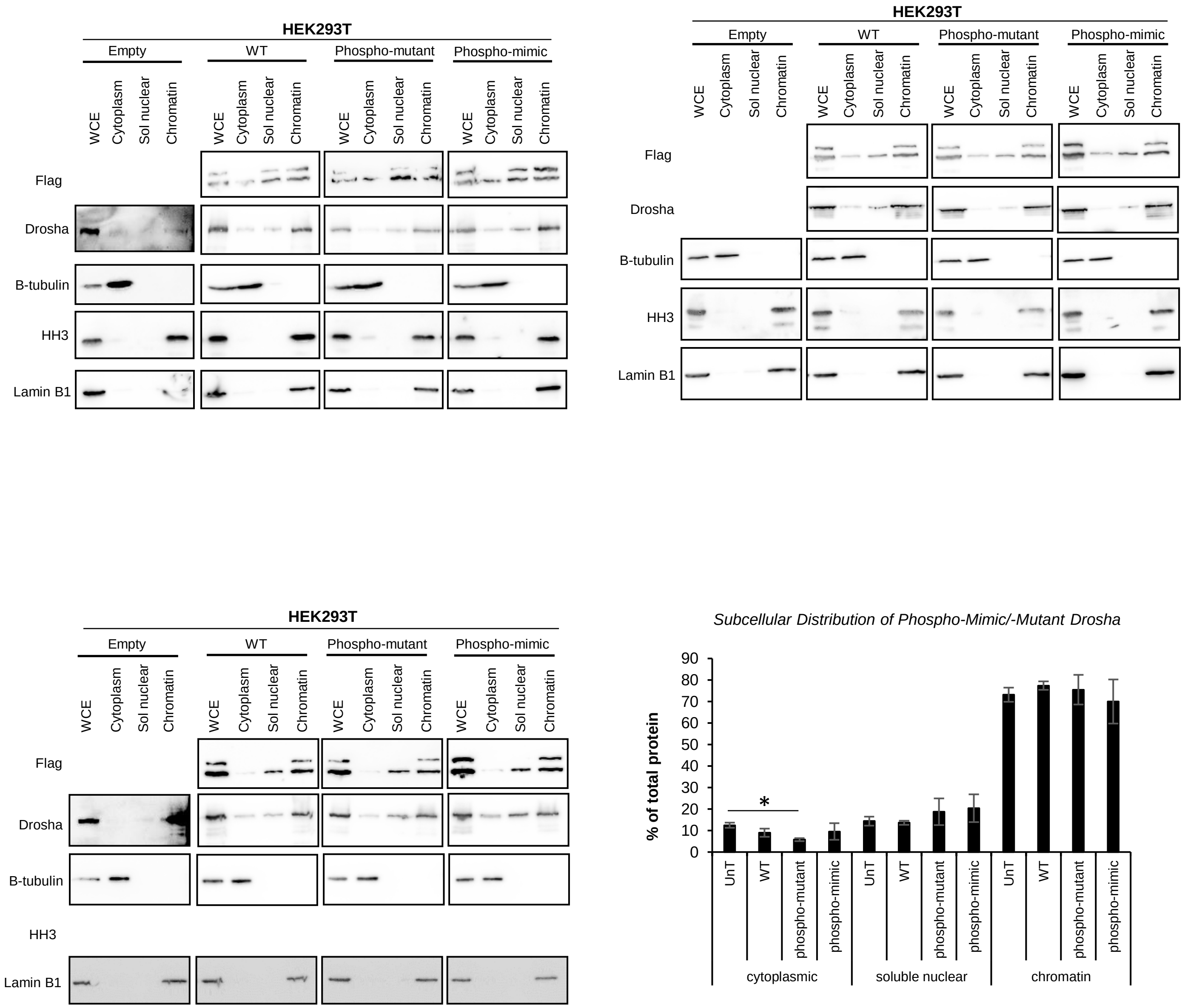

Figure S23: Subcellular Localisation of Wild-Type, Phospho-Mutant ($S^{300}A,S^{302}A$) and Phospho-Mimic ($S^{300}E,S^{302}D$) Flag-Drosha. *A-C)* Western blot analysis of Flag and Drosha protein levels in whole cell extracts (WCEs), cytoplasmic and nuclear fractions of HEK293T cells transfected with WT, phospho-mutant ($S^{300}A,S^{302}A$) and phospho-mimic ($S^{300}E,S^{302}D$) Flag-Drosha for 72h. β-tubulin, Histone H3 and Lamin B were used as cytoplasmic and nuclear controls, respectively. *D)* Densitometry was performed using ImageJ and protein levels shown relative to WCE levels corrected for loading. *Columns:* mean ± SEM for three independent experiments. * $P<0.05$. Data relating to **Fig 5** are shown.

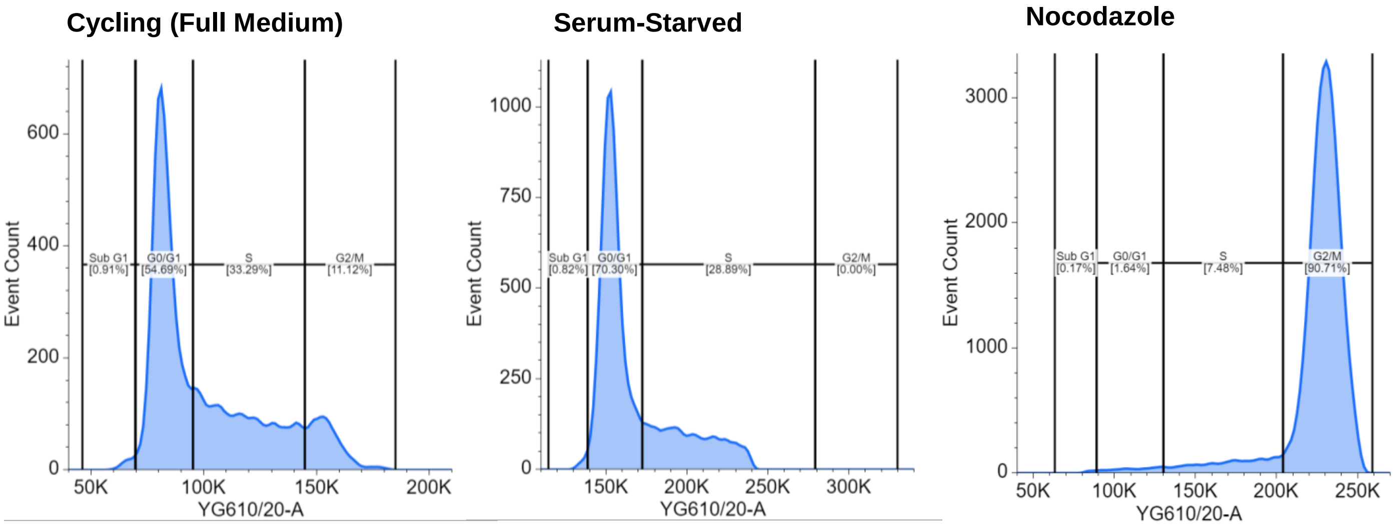

Figure S24: PLK1 Phosphorylation of Drosha at S^{300} and/or S^{302} Alters Drosha:DGCR8 Interaction. Western blot analysis of DGCR8 protein levels in Flag immunoprecipitates of HEK293T cells transfected with WT, phospho-mutant ($S^{300}A$,$S^{302}A$) or phospho-mimic ($S^{300}E$,$S^{302}D$) for 72h. Biological repeats relating to **Fig 5E** are shown.
