# Supplementary material for: PLK1 Regulates MicroRNA Biogenesis through Drosha Phosphorylation
Source: Int J Mol Sci. 2023 Sep 19;24(18):14290. doi: 10.3390/ijms241814290 (PMC10531876; doi:10.3390/ijms241814290)
Supplement: Supplementary file 1 [file ijms-24-14290-s001.zip › Supplementary materials and methods PLK1 miRs paper final.pdf]

Supplementary Materials

qRT-PCR Primers

Amplicon	Forward primer (5' → 3')	Reverse primer (5' → 3')
Pri-miR-93	CACTCCATGTGTCCTAGATCC	CTCGGGAAGTGCTAGCTCAG
Pri-17/18a/19a/20a/19b-1/92a-1	CCAGTCAGAATAATGTCAAAGTG	CAACATCAGCAGGCCCTGC
Pri-miR-1248	CTGTCTGATTGTTAGACATTA	GCTGTTACTTTTCTTCTTGTG
Pri-miR-509	GCTGCCATCCTCAGACATGC	ATGCAGTACTCTACCCACAGAC
Pri-miR-152	GCTGATAGCGCAGGTCCAGCC	GGTCCTTCCGGGCCCAAGTTC
Pri-miR-29c	CTCTTACACAGGCTGACCGA	ACCACAGGCTGCTGCTGC
Pri-miR-892a	GCAGTGCCTTACTCAGAAAG	GACAACTCCTGCAGCTCCA
Pri-miR-21	GTACCACCTTGTCGGGTA	GATGGTCAGATGAAAGATACC
Pri-miR-222/221	TGCAGTAGGCAGTTGTGTTG	GAGAACATGTTTCCAGGTAGC
Pri-miR-34a	CCGTGGACCGGCCAGCTGT	TCTTCCCTCTTGGGCCCCACA
Pri-miR-26a	AGGCCCTGGCGAAGGCCGTG	CGGATGCCGGCGTCCAGGC
Pri-miR-106b/93/25	CAGCAGGGCACGCACAGC	GTCCAAGACGGGAGGACAG
Pri-miR-301b/130b	CTAGACAGGCCTGGGCCTCTG	GTCGGGCAGGCCTGCCAGT
L19	GCGGAAGGGTACAGCCAAT	AGCAGCCGGCGCAAA
Pre-miR-93	CTGGGGGCTCCAAAGTGCTG	CTCGGGAAGTGCTAGCTCAG
Pre-miR-17	GTCAGAATAATGTCAAAGTGCTT	GTCACCATAATGCTACAAGTG
Pre-miR-1248	ACCTTCTTGTATAAGCACTGTG	GCTGTTACTTTTCTTCTTGTG
5S rRNA	TACGGCCATACCACCCTGA	GGCGGTCTCCCATCCAA

Supplementary Methods

Small RNA-Sequencing and Bioinformatics Analysis

QC, small RNA sequencing and bioinformatics analysis was conducted by Novogene. RNA integrity and quantitation were assessed using the RNA Nano 600 Assay Kit on the Bioanalyser 2100 system (Agilent Technologies). 1µg total RNA per sample was used as input and sequencing libraries prepared using NEBNext® Small RNA Library Prep Set for Illumina® (NEB, USA) according to the manufacturer's instructions. Library quality was assessed sequenced using the Ilumina Bioanalyser 2100 system (Agilent Technologies). Libraries were sequenced on Illumina NovaSeq 6000 platform to a minimum depth of 10 million read pairs per sample, and single-end 50bp reads generated. Clean reads were obtained from raw reads in FASTQ format through removal of adapters, poly-N sequences and low-quality reads. Clean reads were mapped to reference genome (GRCh38) using Bowtie. Mapped reads were compared to known miR sequences from miRbase. Expression of known and unique miRNAs in each sample were normalized to TPM (transcripts per million reads). Differential expression analysis was performed using DESeq2 R package. Resultant P values were False Discovery Rate (FDR)-adjusted using the Benjamini and Hochberg's approach. Gene Ontology (GO) and Kyoto Encyclopedia of Genes and Genomes (KEGG) Pathway enrichment analyses were performed using the clusterProfiler R package.
